# Mapping the Global Distribution of *Babesia* Infections

**DOI:** 10.1155/tbed/5889219

**Published:** 2025-11-24

**Authors:** Bo-Kang Fu, Tian Tang, Ming Yue, Jin-Jin Chen, Hong Su, Xiao-Bin Huang, Yu-Feng Yang, Simon I. Hay, Li-Qun Fang, Wei Liu

**Affiliations:** ^1^School of Public Health, Anhui Medical University, Hefei 230022, China; ^2^State Key Laboratory of Pathogen and Biosecurity, Academy of Military Medical Science, Beijing 100071, China; ^3^Center for Disease Control and Prevention of Central Theater Command, Beijing 100042, China; ^4^Department of Infectious Diseases, The First Affiliated Hospital with Nanjing Medical University, Nanjing 210029, China; ^5^Department of Health Metrics Sciences, School of Medicine, University of Washington, Seattle, Washington, USA; ^6^Institute for Health Metrics and Evaluation, University of Washington, Seattle, Washington, USA; ^7^Department of Infectious Diseases, The First Affiliated Hospital of Anhui Medical University, Hefei 230022, China

## Abstract

Understanding the ecological niches and quantifying the disease burden of *Babesia* species is essential for efficient surveillance and control strategies. Through a systematic review of global distributions, we document all 250 identified *Babesia* species across 73 vector species, 224 animals, and humans. *Babesia caballi* infected the broadest range of tick species, while *Babesia microti* exhibited the highest prevalence in wildlife. Among 26 848 recorded human cases involving 10 *Babesia* species, >90% were attributed to *Babesia microti* and *Babesia duncani*. Using three machine learning algorithms, we evaluated ecological and vector-associated determinants governing the distributions of six predominant *Babesia* species. Our models predict *B. bovis* to have the most extensive geographic range. Critically, habitat suitability index (HSI) of vector ticks emerged as the primary driver of *Babesia* transmission risk. Enhanced awareness, diagnostic capacity, and surveillance are imperative in identified high-risk regions.

## 1. Introduction

Babesiosis, caused by intraerythrocytic protozoa belonging to the genus *Babesia* within the family Babesiidae of suborder Piroplasmida, ranks among the most prevalent tick-borne diseases, with great economic, veterinary, and medical impact worldwide [1, 2]. Its clinical spectrum ranges from asymptomatic infection to severe illness, occasionally resulting in mortality [3]. Fatality rates among hospitalized patients have been reported at 1.6% and can escalate to as high as 21.4% in immunocompromised individuals [4, 5]. Transmission to humans primarily occurs through the bite of infected ticks, with occasional cases caused by blood transfusion and rare instances of perinatal transmission [6–8]. *Babesia* species are classified morphologically into small forms (<2.5 *μ*m in diameter; e.g., *B. microti*, *B. divergens*) producing tetraploid merozoites, and large forms (>2.5 *μ*m in diameter; e.g., *B. bovis*, *B. bigemina*) generating diploid merozoites via budding [2]. Diagnostic ambiguity with malaria persists due to morphological similarity to *Plasmodium* in blood smears and serological cross-reactivity [9].

In recent years, the geographic range of *Babesia* has expanded globally, driven by climate-mediated tick expansion, unconstrained livestock and wildlife movements, and inconsistent tick control measures, thus altering the epizootiology of the disease [10]. Ixodid ticks, specially *Ixodes ricinus* and *Dermacentor reticulatus*, serve as the primary vectors for *Babesia*, capable of infecting various vertebrate mammals, including humans [11, 12]. In the United States, national surveillance data reveal >22,000 cases reported during 2011–2023, with endemic states expanding from 7 to 13 [13]. Canada reports a parasite prevalence of approximately one in 5000 black-legged tick (*Ixodes scapularis*) in 2020 [14]. The expanding geographic distribution is not limited to national borders but also poses a transboundary threat facilitated by human mobility. International travel has emerged as a significant pathway for the importation of babesiosis from endemic to nonendemic regions, as evidenced by multiple recent cases associated with international travel [15, 16]. This trend underscores the necessity of a global perspective on *Babesia* surveillance and risk assessment.

In the recent decade, advances in microscopy, cell biology, and molecular biology techniques, have facilitated the discovery of >100 *Babesia* species, rapidly expanding our knowledge of the *Babesia*. However, current research on geographic distribution and risk assessment of *Babesia* infection remains largely confined to national or administrational level, or focuses on a single-species analysis. Consequently, the global distribution pattern and associated disease burden of various *Babesia* species remained poorly characterized.

This study presents a comprehensive literature review of *Babesia* infection's global distribution. We examine infection diversity of *Babesia* across vectors, animal hosts, and human populations, alongside their geographic distribution patterns. Furthermore, we established ecological machine learning models at 10 km× 10 km resolution to predict potential risk areas for six major *Babesia* species, with results that could enhance global babesiosis surveillance.

## 2. Materials and Methods

### 2.1. Data Compiling

We conducted a comprehensive literature search in PubMed, Web of Science, bioRvix, and medRvix for articles published between 1957 and 2022, using the search terms “*Babesia*” or “Babesiosis,” without language restriction. The GenBank database were also searched using “*Babesia*” to identify all sequenced *Babesia* species. Initial screening involved title/abstract evaluation, followed by full-text article reviews by two authors (Bo-Kang Fu and Jin-Jin Chan). Studies were included if they described laboratory-confirmed *Babesia* detection in arthropod vectors, animals, or humans, from natural infections rather than experimental challenges. To identify potentially missed studies, we repeated searches for each *Babesia* species using their taxonomic names. Exclusion criteria comprised: lack of laboratory confirmation or definitive species identifications; unclear sampling origin/location; drug/vaccine trials; laboratory-based transmission studies (Supporting Information 1: Table S1). The protocol was registered with PROSPERO (ID: CRD42024529771) and conducted according to the Preferred Reporting Items for Systematic Reviews and Meta-Analyses (PRISMA) guideline (Supporting Information 2). Confirmed infections were determined based on microscopy findings, molecular assay based on over two amplification targets, or pathogen isolation (Supporting Information 1: Table S2) [13].

### 2.2. Data Extraction and Spatial Standardization

From each validated study, we extracted 14 variables across eight baseline information categories (Supporting Information 3), including host types (vector, animal, or human being), total number of detections and number of positive detection (stratified by host type), and human clinical manifestations (Supporting Information 1: Table S3). The classification of *Babesia* species as “confirmed” in this study was determined according to the official taxonomic framework provided by the National Center for Biotechnology Information (NCBI) Taxonomy database. A species was considered as “confirmed” only if it was formally recognized within the genus *Babesia* and assigned a valid taxonomic status (e.g., *Babesia microti*). Entries labeled as “unclassified *Babesia*” were excluded from the “confirmed” category to ensure the accuracy and reliability of subsequent analyses. Clinical symptoms analysis was restricted to confirmed human cases. We additionally recorded detections of multiple *Babesia* species in individual ticks to illustrate their coinfections. For the risk analysis of *Babesia* infection, we integrated 44 environmental, ecoclimatic, biological, and socioeconomic variables selected a priori based on their known association with vectors or transmission (Supporting Information 1: Tables S5–S7) [17–20]. These variables were standardized to a global 10 km× 10 km grid, with mean values calculated per grid cell (Supporting Information 3).

### 2.3. Occurrence Location Definition and Processing

For each *Babesia* species, a “positive occurrence location” was defined as ≥1 confirmed *Babesia* infection at unique geographic coordinates, regardless of host type or detection period. Where precise coordinates were unavailable, the smallest administrative unit (county, city, or province) served as a polygon representation. Centroid coordinates for these polygons were derived from authoritative geospatial databases (primarily Google Maps). All locations underwent rigorous geospatial validation to ensure accuracy and remove duplicates. To minimize ecological modeling bias, we applied an area exclusion criterion: records lacking specific coordinates or represented by polygons exceeding 100 km^2^ (local scale) and 400 km^2^ (regional scale) were excluded.

### 2.4. Niche Modeling of the Six Major *Babesia* Species

Among the 10 *Babesia* species confirmed to infect humans, we identified six major *Babesia* species based on their occurrence in ≥80 positive grid cells (Supporting Information 1: Table S8): *Babesia microti*, *Babesia* sp. venatorum, *Babesia divergens*, *Babesia bigemina*, *Babesia bovis*, and *Babesia odocoilei*. Primary vector tick species for these major *Babesia* species were determined through a meta-analysis of detection data and literature reviews (Supporting Information 1: Table S9; Supporting Information 3) [21, 22], supplemented by the Global Biodiversity Information Facility (GBIF) [23] and VectorMap database (Supporting Information 1: Table S10; Supporting Information 4).

The habitat suitability index (HSI) for each tick species was estimated as previously described [17, 24]. Environmental, ecoclimatic, biological, and socioeconomic variables were integrated into niche models for the six *Babesia* species. For the data preparation, tick occurrence coordinates were mapped to a 10 km× 10 km global grid to associate with ecological variables. Occurrence grids served as “case” points. Pseudo-absence grids were randomly sampled from areas located >30 km from each presence locations cell at a ratio of 3:1 [25]. Subsequently, a boosted regression trees (BRT) model was employed to predict the probability of occurrence for each tick species by using environmental, ecoclimatic, and biological variables as predictors. The modeling process was repeated 100 times to reduce random effects. The mean predicted value across all iterations represented the HSI for each tick species [17, 24]. HSIs for three tick species, namely *Ixodes persulcatus*, *Ixodes ricinus*, and *Ixodes scapularis*, have already been predicted in previous studies and were directly used in this study [25]. Subsequent internal validation was conducted using the relative uncertainty, which is defined as the ratio of the 95% uncertainty interval to the HSI.

In the second phase, we conducted niche modeling for the six major *Babesia* species to quantify relationships between occurrence risks and influencing factors, and to predict potential high-risk areas (Supporting Information 5). Three machine learning algorithms were employed with 10-fold block cross-validation: BRT, random forest (RF), and Least Absolute Shrinkage and Selection Operator (LASSO) logistic regression [26]. To avoid overfitting and to improve interpretability of the models, we first screened for multicollinearity among candidate predictors. We used the R package “usdm” to calculate the variance inflation factor (VIF) and exclude variables with a VIF greater than 10 (Supporting Information 1: Table S16). We then fitted an initial model for each species, and predictors with relative contributions (RCs) greater than 3% were retained for the formal modelbuilding [17, 25]. Predictive performance was assessed using the mean (95% CI) of RCs and area-under-curve (AUC) of receiver operating characteristic (ROC) curves computed across100 model iterations. We further compared pseudo-absence sampling methods (random grids vs. background grids) in higher-performing models (AUC >0.8) selecting the approach yielding the highest mean AUC as final (Supporting Information 3). By integrating population data with predicted *Babesia* habitat suitability, we estimated both the size of at-risk human populations and the geographic extent of areas vulnerable to these six major *Babesia* species.

## 3. Results

### 3.1. Literature Screening and Study Inclusion

**Figure 1:**
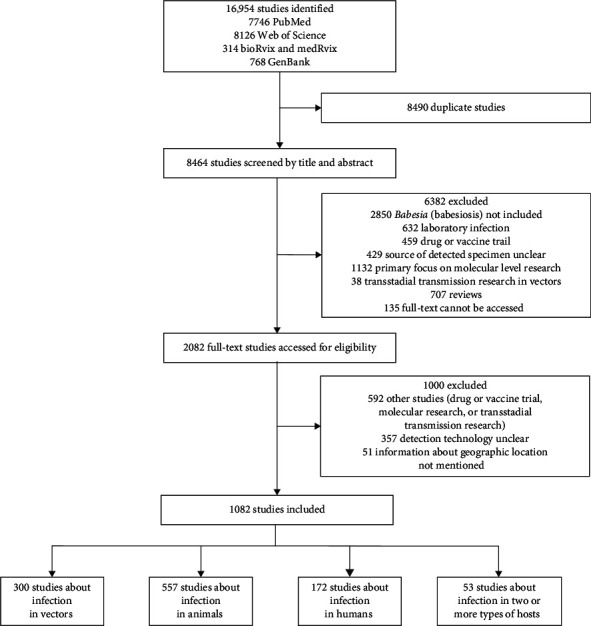
The flow diagram of literature review and data collection.

Our systematic review identified 8464 unique publications from global databases. After rigorous screening, 7382 studies were excluded, yielding 1082 (12.8%) articles, supplemented by human babesiosis surveillance data obtained from the Centers for Disease Control and Prevention (CDC) in the United States, were used for final analysis (Supporting Information 6). The included reports comprised 557 (51.5%) publications documenting *Babesia* detection in animals; 300 (27.8%) documenting infection in vectors, 172 (15.9%) concentrating on human infections; 53 (4.9%) reporting *Babesia* detection in at least two types of hosts, primarily involving vectors and animal hosts (Figure 1).

### 3.2. Temporal Expansion of *Babesia* Diversity

**Figure 2:**
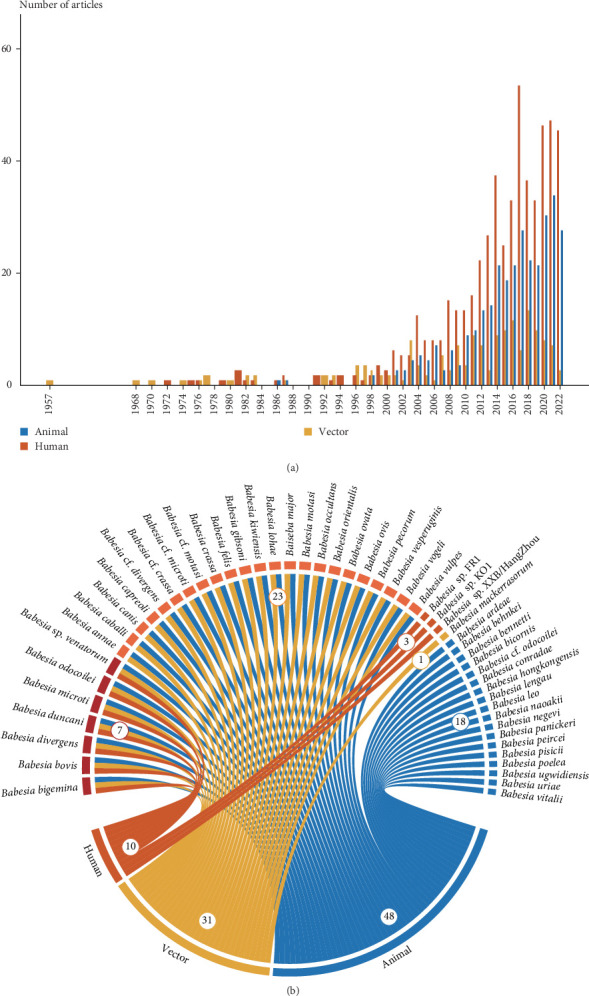
Publications about *Babesia* in animals, vectors, and human beings. (A) Annual number of publications on *Babesia* stratified by host type. (B) Chord diagram depicting association between *Babesia* species and host types.

Since the first human case in 1957 [27], there has been an increasing number of reports on more *Babesia* species in animals, vectors, and humans (Figure 2A). According to the Taxonomy Browser of the NCBI, we cataloged 250 distinct *Babesia* species, among which only 48 were validated *Babesia* species (Supporting Information 1: Table S4). Four previously unclassified pathogens (*Babesia* sp. FR1, *Babesia* sp. KO1, *Babesia* sp. venatorum, and *Babesia* sp. XXB/HangZhou) demonstrated human pathogenicity, and were included in subsequent analyses. Overall, 10 *Babesia* species were identified to infect humans, 31 were harbored by arthropod vectors, 48 were detected in animal hosts, and seven were linked to all three hosts (Figure 2B).

### 3.3. Vector Associations With *Babesia* Species

**Figure 3:**
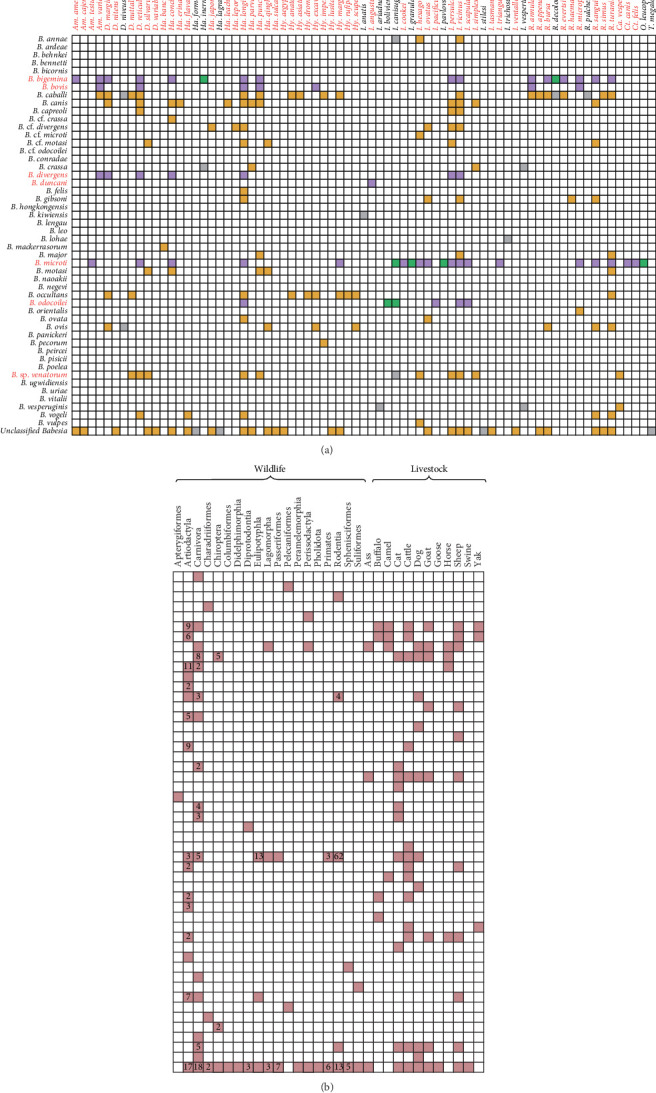
The detection of *Babesia* species in vectors (A) and animals (B). The *Babesia* species that infect humans are highlighted in red fonts. Tick vectors known to bite humans are marked in red. The purple square denotes pathogenic *Babesia* species transmitted by vectors that bite humans; green square denotes pathogenic *Babesia* species transmitted by vectors that do not bite humans; orange square denotes nonpathogenic *Babesia* species carried by vectors that bite humans; gray square denotes *Babesia* species carried by vectors that do not bite humans. The number within each square represents the count of wild animal species involved. The abbreviations in figure A denote: Am, *Amblyomma*; Ca, *Carios*; Ct, *Enocephalides*; D, *Dermacentor*; Ha, *Haemaphysalis*; Hy, *Hyalomma*; I, *Ixodes*; O, *Orchopeas*; R, *Rhipicephalus*; T, *Tabanus*.

A total of 73 arthropod species were identified as carrier of *Babesia*, primarily comprising 69 tick species (58 hosted 31 validated *Babesia* species, while 11 carried only unclassified); supplemented by three flea species, and one fly species. *Babesia caballi* was identified in the broadest vector range (20 tick species), followed by *Babesia microti* (18 tick species), *Babesia bigemina* (17 tick species), and *Babesia canis* (12 tick species), all of which were hard ticks. *Babesia* sp. venatorum and *Babesia vesperuginis* were carried by both hard ticks and soft ticks. Within all tick vectors, *Ixodes* and *Haemaphysalis* genera harbored the greatest variety of *Babesia*, with 18 *Ixodes* species and 12 *Haemaphysalis* species each carrying 21 *Babesia* species. This was followed by *Rhipicephalus* genus (11 species carrying 13 *Babesia* species), *Hyalomma* (eight carrying six *Babesia* species), *Dermacentor* (five carrying 13), *Amblyomma* (three carrying five), and *Carios* (one carrying two). Among vectors that could carry *Babesia*, a total of 54 tick species and two flea species were recorded with human-biting history (Figure 3A) (Supporting Information 1: Table S11), which warrant further medical attention. Nine *Babesia* species exhibited coinfections within four tick vectors (*Dermacentor reticulatus*, *Haemaphysalis punctata*, *Ixodes ricinus*, and *Ixodes scapularis*) (Supporting Information 1: Table S12).

### 3.4. Animal Host Association With *Babesia* Species

A total of 48 *Babesia* species were documented infecting 159 animal species, comprising 38 *Babesia* species carried by 149 species of wildlife hosts and 28 *Babesia* species carried by 10 domestic animals (Figure 3B). Notably, *Babesia microti* showed the broadest host range among wildlife (88 species), followed by *Babesia canis* and *Babesia capreoli* (each in 13 species). In contrast, among domestic animals, *Babesia bigemina*, *Babesia caballi*, and *Babesia gibsoni* infected the greatest number of domestic animals (six species for each), followed by *Babesia bovis*, *Babesia canis*, and *Babesia vogeli* (five each). The primary hosts belonged to the orders Artiodactyla (24 species, including *Cervus elaphus*, *Capreolus pygargus*, and *Dama dama* hosting 17 *Babesia* species), Carnivora (21 species, including *Vulpes vulpes*, *Panthera leo*, and *Acinonyx jubatus* hosting 16 *Babesia* species), Rodentia (65 species, including *Apodemus agrarius*, *Apodemus speciosus*, and *Myodes rufocanus* hosting four *Babesia* species), and Eulipotyphla (14 species including *Neotetracus sinensis*, *Blarina brevicauda*, and *Sorex araneus* hosting two *Babesia* species). Additionally, 53 wild animals and two domestic animal species were exclusively infected by unclassified *Babesia*.

### 3.5. Geospatial Distribution Patterns of Babesiosis

**Figure 4:**
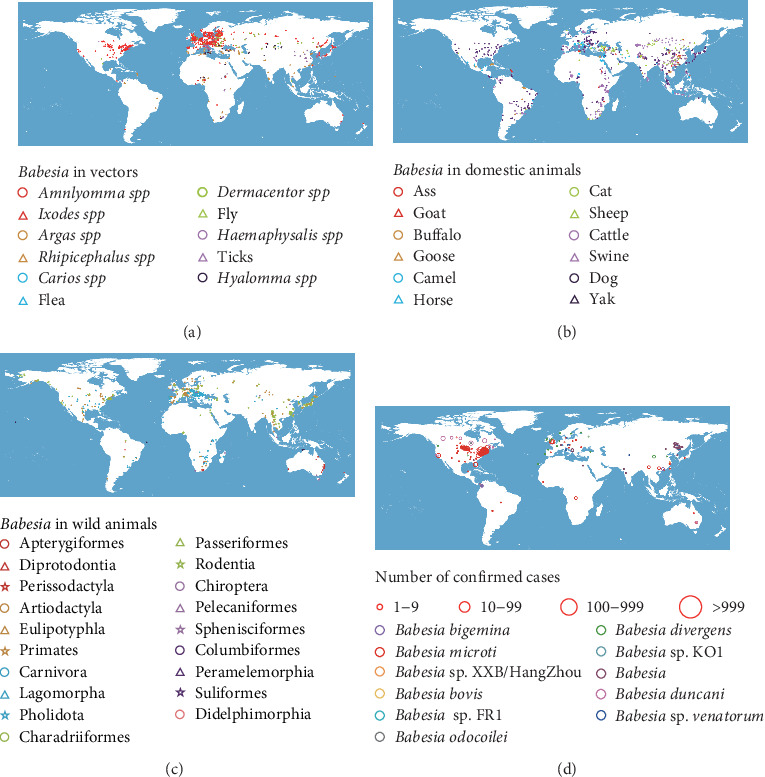
Spatial distributions of *Babesia* detection events. (A) Distribution of vectors detecting *Babesia* with ticks classified by genus. (B) Distribution of domestic animals detecting *Babesia* classified by common name. (C) Distribution of wild animals detecting *Babesia* classified by order. Different colors and shapes represent different types of hosts. (D) Distribution of humans detecting *Babesia*. The size of the red circles represents the number of people at that location.

A total of 1922 records of *Babesia* infection in arthropods were identified (1918 in ticks, three in fleas, and one in fly), which revealed distinct spatial clustering pattern that varied by latitude or continent. *Ixodes*-associated infections predominated (*n* = 1227), concentrated in Northern Hemisphere temperate zones, particularly Europe and the northeastern United States. *Dermacentor* infections (*n* = 153) clustered in high-latitude Eurasian regions, while *Rhipicephalus* records (*n* = 154) showed no significant spatial patterning (Figure 4A).

A total of 2911 records of *Babesia* infection in animals (1452 records in wild animals and 1459 records in domestic animals) were identified. Amont them livestock infections were globally widespread, with cattle (*n* = 618) and dogs (*n* = 407) representing the most affected species (Figure 4B). Wildlife infections peaked in Rodentia (*n* = 437) and Carnivora (*n* = 428), occurring across all inhabited continents except Oceania. Artiodactyla infections clustered in North America, the Mediterranean region, and East Asia (Figure 4C). A total of 26,848 confirmed human infections with *Babesia* were recorded, predominantly in North America, Europe, and East Asia (Figure 4D). *B. microti* caused 95.3% of cases (*n* = 25,576), followed by *B. duncani* (4.2%, *n* = 1133), *Babesia* sp. venatorum (0.2%, *n* = 58), and *B. bigemina* (0.2%, *n* = 48). Transfusion transmission accounted for 226 cases (0.8%), exclusively involving *B. microti* and *B. duncani*. Clinical records were available for 706 patients, including 622 cases of *Babesia microti* infection, 40 cases of *Babesia* sp. venatorum infection, and 29 cases of *Babesia divergens* infection. Cases with fewer than 10 clinical reports were excluded from the analysis (Supporting Information 1: Table S13). Based on these data, the most common symptoms of babesiosis were fever (77.7%) and fatigue (54.6%), followed by chills (46.3%), sweats (36.5%), and anorexia (30.1%) (Supporting Information 1: Table S14; Supporting Information 2).

**Figure 5:**
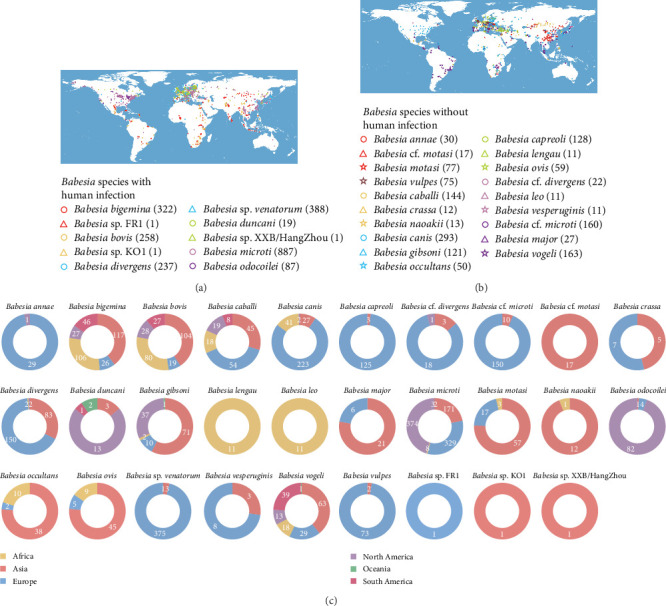
Spatial distributions of 29 *Babesia* species. (A) 10 *Babesia* species with confirmed human infection. (B) About 19 *Babesia* species without recording human infection. The circles of different colors represent different species of *Babesia* parasites. (C) Number of occurrence locations for totally 29 *Babesia* species, stratified by continent. For each *Babesia* species, a positive occurrence location refers to one or more confirmed cases of *Babesia* infection at a distinct geographic site with defined coordinates, irrespective of the host type or detection time.

Our spatial mapping of 10 human-pathogenic *Babesia* species revealed distinct biogeographical patterns. Human infections with *B. venatorum* have been exclusively documented in Eurasia, while *B.divergens* predominantly found in this region (Figure 5A). Analysis of 19 *Babesia* species ≥10 documented occurrence sites demonstrated significantly greater *Babesia* diversity and abundance across Eurasian territories (Figure 5B). *Babesia microti* and *Babesia vogeli* exhibit the broadest distribution, having been reported across all six continents. Four additional *Babesia* species (*Babesia bigemina*, *Babesia bovis*, *Babesia caballi*, and *Babesia gibsoni*) were confirmed within five inhabited continents (Figure 5C). Distribution of remaining *Babesia* were detailed in Supporting Information 1: Figure S1.

### 3.6. Ecological Niche Modeling

Our risk prediction analysis focused on two predominant tick species (*Dermacentor reticulatus* and *Rhipicephalus microplus*, revealing tick species-specific drivers of habitat suitability (Supporting Information 1: Table S15; Figures S2–S5; Supporting Information 1). Using HSI modeling, we mapped potential distributions for each species within their endemic regions. For the six major *Babesia* species, RF models demonstrated superior predictive performance compared to BRT and LASSO. Optimized RF performance was achieved at 400 km^2^ spatial resolution and through random sampling strategies, when compared with a 100 km^2^ threshold and background sampling, as evidenced by higher average AUC (Supporting Information 1: Tables S17,18; Figure S6). Crucially, vector tick HSI emerged as the strongest predictor for all six major *Babesia* species occurrence (Supporting Information 1: Table S19), though secondary drivers varied: socioeconomic variables were associated with the occurrence of all six *Babesia* species; ecoclimatic factors were significantly associated with the distribution of *Babesia* sp. venatorum; cattle density positively correlated with the occurrence of *Babesia bigemina* (Supporting Information 1: Figures S7–S12).

By overlaying population data onto suitable habitats for *Babesia* distribution, we quantified exposure risks across endemic zones for each of the six major *Babesia* species (Table 1). *Babesia bovis* showed the most extensive geographic distribution (25.2 million km^2^), followed by *Babesia bigemina* (20.0 million km^2^), and *Babesia microti* (14.5 million km^2^). Collectively, the predicted risk areas of *Babesia* infection substantially exceed previously documented ranges (Supporting Information 1: Figures S13–S18), indicating a widespread underdetection of babesiosis-related threats.

## 4. Discussion

Babesiosis is increasingly recognized as a globally significant public health threat, posing substantial risks to livestock and companion animal health while incurring considerable economic costs worldwide [28]. Our analysis provides a comprehensive overview of reported *Babesia* species based on classical taxonomic criteria, confirming the *Babesia* infections in at least 69 tick species, while other arthropods, such as fleas, play a limited role in *Babesia* ecology. Historically, hard ticks (Ixodidae) were considered the primary vectors; however, recent molecular evidence has detected *Babesia* DNA in soft ticks (Argasidae) and nontick vectors, suggesting a broader range of potential vectors requires further investigation [29, 30]. Among confirmed hard tick vectors, the genera *Ixodes* and *Haemaphysalis* harbor the highest diversity of *Babesia* species, which might be associated with the broad host range that can spread the *Babesia* species.

Notably, *Babesia* sp. venatorum has been detected in both hard and soft ticks. Its transmission by *I. ricinus* through transovarial and transstadial routes has been well-documented, highlighting the critical role of these tick species in disease incidence [31, 32]. Emerging evidence suggests changing distribution of ticks, attributable to climate change and anthropogenic factors [33], exemplified by the northward expansion of *I. scapularis* in Canada and the corresponding rise in babesiosis cases in the affected areas [34]. Current models further predict range expansion for *Dermacentor reticulatus* and *Rhipicephalus microplus*, amplifying potential risk zones for babesiosis. These trends highlight the imperative for enhanced tick surveillance systems to provide early alerts for emerging babesiosis outbreaks [35].

Given the extensive diversity of species reported as *Babesia* hosts, all vertebrates could be potential carriers provided they are suitable hosts for *Babesia*-vector ticks [2]. To date, we identified over 100 *Babesia* species in both wild and domestic animals. Established natural transmission cycles include *B. microti* circulating between *I. scapularis* ticks and white-footed mice in North America, as well as *B. divergens* circulating between *I. ricinus* ticks and bovine hosts in Europe [11, 36, 37]. Furthermore, vertical transmission of tick-borne *Babesia microti* has been observed in its natural host *Peromyscus leucopus* [38]. Additionally, migratory birds contribute to geographic dissemination of *Babesia* infection into nonendemic areas, which has been acknowledged [39]. Larval ticks that attach to and feed on infected birds can be transported over long distances, potentially establishing new endemic over hundreds of miles [20, 40]. Collectively, avian migration, vector ecology, animal reservoirs, and human activities accelerate *Babesia* spread from high- to low-risk regions.

Although tick bites represent the primary transmission route, over 200 cases of transfusion-transmitted babesiosis cases have been reported in endemic areas. However, this figure likely underestimates the true disease burden, particularly in endemic regions where blood screening for *Babesia* infection among blood donors is not implemented and clinical awareness remains limited. Systematic evidence confirms *Babesia microti* as the predominant *Babesia* species in human infection [12, 41, 42], also representing the most prevalent *species* transmitted via blood transfusion, accounting for > 95% of transfusion-transmitted cases [7, 43]. Moreover, *Babesia duncani* has also caused transfusion-related infections [7, 44]. Given the life-threatening risk to immunocompromised recipients, clinicians in endemic regions should consider babesiosis in the differential diagnosis of febrile illness.

Our ecological niche modeling of all the six major *Babesia* revealed that the HSI of vector ticks is the strongest predictor of infection risk. For *B. microti*, GDP and urbanization rate significantly influence its occurrence, likely reflecting both its high prevalence in developed regions, and enhanced surveillance efforts for detecting *Babesia* species. Climate factors was also demonstrated to shape the distribution of *Babesia* sp. venatorum, which occurs predominantly in Europe, but also been in the dry and cold climates of Northeastern China [45]. The projected risk maps should guide targeted surveillance in data-limited high-risk regions.

This study has several inherent limitations. First, the data were derived from study reports, potentially introducing bias due to inconsistency in the quality of *Babesia* infection detection and reporting across different countries and regions. Second, genotype-specific information is rarely reported in the literature included in this study, precluding genotype-level analysis. Given that distinct genotypes of *B. microti* (e.g., the U.S.-type and Kobe-type) have been identified and some are confirmed to cause human disease [46], the potential impact of different genotypes on disease outcome was not addressed in this study and warrants further investigation. Third, in resource-limited settings, babesiosis might be misdiagnosed as malaria or other clinically similar diseases, resulting in an underestimation of *Babesia* infections. In addition, debate persists regarding the taxonomical classification and confirmed human pathogenicity of specific *Babesia* species, which could affect the reliability of their mapping and modeling analysis.

Despite these limitations, this study establishes a comprehensive up-to-date database and thematic maps detailing the global distribution of *Babesia* species. We have identified the major *Babesia* species, their competent vectors, animal hosts, along with their association, enabling the prediction of regions at risk for these pathogens. This work substantially enhanced our understanding of babesiosis distribution and ecology and bolsters surveillance capabilities.

## Figures and Tables

**Table 1:** Average AUC, areas, and population sizes predicted by the RF models at potentially medium-to-high risk of exposure to the six major *Babesia* species.

*Babesia* species	Average testing AUC (95% CI)	Population size (million)						Area (10,000 km^2^)					
		Europe	Asia	Africa	Americas	Oceania	Worldwide	Europe	Asia	Africa	Americas	Oceania	Worldwide
*B. microti*	0.936 (0.912‒0.954)	260.9	833.5	17.5	169.2	1.6	1282.7	801.1	242.2	1.8	404.2	0.7	1450.0
*B*. sp. venatorum	0.975 (0.963‒0.977)	387.0	36.1	—	—	—	423.1	1479.6	24.3	—	—	—	1503.9
*B. bigemina*	0.921 (0.865‒0.956)	10.6	256.8	415.6	208.2	2.9	894.1	27.7	141.8	1195.3	623.5	8.0	1996.3
*B. bovis*	0.917 (0.859‒0.955)	176.0	2096.0	603.1	634.2	23.9	3509.3	211.1	1024.6	595.6	631.0	52.8	2515.1
*B. divergens*	0.984 (0.966‒0.995)	432.6	209.2	13.6	—	—	655.5	1830.2	386.0	3.6	—	—	2219.8
*B. odocoilei*	0.959 (0.911‒0.996)	—	—	—	144.1	—	144.1	—	—	—	920.3	—	920.3

## Data Availability

The data that supports the findings of this study are available in the Supporting Information.

## Supporting Information

Supporting Information 1 Table S1: the inclusion and exclusion criteria of screening publications. Table S2: the laboratory tests used to detect *Babesia* infections in the reviewed studies. Table S3: list of variables extracted from reviewed studies. Table S4: the references for all the *Babesia* species. Table S5: the spatial resolution, study duration, and source of the included data. Table S6: ecological factors potentially associated with the ticks and tick pathogens used in the modeling analysis. Table S7: variables used for ecological modeling in this study. Table S8: the number of studies and positive occurrence grids for the ten *Babesia* species with reports of confirmed human infection. Table S9: the tick species carrying the six major *Babeisa* species. Table S10: the number of studies and occurrence locations for the two tick species. Table S11: vectors with evidence of biting human. Table S12: the coinfection of *Babesia* species and their infected vectors. Table S13: the number of human cases of *Babesia* infection. Table S14: clinical characteristics of human infections with *Babesia* species. Table S15: the RCs of significant contributors to the occurrence of two main species of ticks based on BRT models. Table S16: the VIF values for each variable in the models of *Babesia*. Table S17: comparison of AUC values for the models of *Babesia* species using two area thresholds. Table S18: comparison of AUC values for the models of *Babesia* species using two sampling methods. Table S19: the RCs of significant contributors to the spatial distribution of the six major *Babesia* species based on RF models. Figure S1: the distributions of other *Babesia* species. Figure S2: effects of major predictors (RCs >3%) for presence of *Dermacentor reticulatus* based on BRT models. Figure S3: effects of major predictors (RCs >3%) for presence of *Rhipicephalus microplus* based on BRT models. Figure S4: the recorded and predicted distributions of *Dermacentor reticulatus* in Europe. Figure S5: the recorded and predicted distributions of *Rhipicephalus microplus* within global range. Figure S6: predictive performance of the three machine-learning algorithms. Figure S7: effects of major predictors (RCs >5%) for presence of *Babesia microti* based on RF models. Figure S8: effects of major predictors (RCs >5%) for presence of *Babesia* sp. venatorum based on RF models. Figure S9: effects of major predictors (RCs >5%) for presence of *Babesia divergens* based on RF models. Figure S10: effects of major predictors (RCs >5%) for presence of *Babesia bigemina* based on RF models. Figure S11: effects of major predictors (RCs >5%) for presence of *Babesia bovis* based on RF models. Figure S12: effects of major predictors (RCs >5%) for presence of *Babesia odocoilei* based on RF models. Figure S13: the recorded and predicted distributions of *Babesia microti* within global range. Figure S14: the recorded and predicted distributions of *Babesia* sp. venatorum in Eurasia. Figure S15: the recorded and predicted distributions of *Babesia divergens* in Eurasia and Africa. Figure S16: the recorded and predicted distributions of *Babesia bigemina* within global range. Figure S17: the recorded and predicted distributions of *Babesia bovis* within global range. Figure S18: the recorded and predicted distributions of *Babesia odocoilei* in Americas.

Supporting Information 2 PRISMA Checklist.

Supporting Information 3 Supplementary methods and results.

Supporting Information 4 The occurrence grid and data sources of each tick species.

Supporting Information 5 Locations with *Babesia* infection in vectors, animals, and humans used in models.

Supporting Information 6 The titles of the 1082 articles included in the study.
