# Supplementary material for: Mapping the Global Distribution of Babesia Infections
Source: Transbound Emerg Dis. 2025 Nov 24;2025:5889219. doi: 10.1155/tbed/5889219 (PMC12668841; doi:10.1155/tbed/5889219)

**Supplementary Tables and Figures**

[Table S1: The inclusion and exclusion criteria of screening publications. 3](#_Toc211020197)

[Table S2: The laboratory tests used to detect](#_Toc211020198) *[Babesia](#_Toc211020198)* [infections in the reviewed studies. 4](#_Toc211020198)

[Table S3: List of variables extracted from reviewed studies 5](#_Toc211020199)

[Table S4: The references for all the](#_Toc211020200) *[Babesia](#_Toc211020200)* [species. 6](#_Toc211020200)

[Table S5: The spatial resolution, study duration and source of the included data. 15](#_Toc211020201)

[Table S6: Ecological factors potentially associated with the ticks and tick pathogens used in the modelling analysis. 16](#_Toc211020202)

[Table S7: Variables used for ecological modelling in this study. 18](#_Toc211020203)

[Table S8: The number of studies and positive occurrence grids for the ten](#_Toc211020204) *[Babesia](#_Toc211020204)* [species with reports of confirmed human infection. 20](#_Toc211020204)

[Table S9: The tick species carrying the six major](#_Toc211020205) *[Babeisa](#_Toc211020205)* [species. 21](#_Toc211020205)

[Table S10: The number of studies and occurrence locations for the two tick species. 23](#_Toc211020206)

[Table S11: Vectors with evidence of biting human 24](#_Toc211020207)

[Table S12: The co-infection of](#_Toc211020208) *[Babesia](#_Toc211020208)* [species and their infected vectors. 27](#_Toc211020208)

[Table S13: The number of human cases of](#_Toc211020209) *[Babesia](#_Toc211020209)* [infection](#_Toc211020209)*[.](#_Toc211020209)* [28](#_Toc211020209)

[Table S14: Clinical characteristics of human infections with](#_Toc211020210) *[Babesia](#_Toc211020210)* [species. 29](#_Toc211020210)

[Table S15: The RCs of significant contributors to the occurrence of two main species of ticks based on BRT models. 30](#_Toc211020211)

[Table S16: The VIF values for each variable in the models of](#_Toc211020212) *[Babesia](#_Toc211020212)*[. 31](#_Toc211020212)

[Table S17: Comparison of AUC values for the models of](#_Toc211020213) *[Babesia](#_Toc211020213)* [species using two area thresholds. 33](#_Toc211020213)

[Table S18: Comparison of AUC values for the models of](#_Toc211020214) *[Babesia](#_Toc211020214)* [species using two sampling methods. 34](#_Toc211020214)

[Table S19: The RCs of significant contributors to the spatial distribution of the six major](#_Toc211020215) *[Babesia](#_Toc211020215)* [species based on RF models. 35](#_Toc211020215)

[Figure S1: The distributions of other](#_Toc211020216) *[Babesia](#_Toc211020216)* [species. 37](#_Toc211020216)

[Figure S2: Effects of major predictors (RCs >3%) for presence of](#_Toc211020217) *[Dermacentor reticulatus](#_Toc211020217)* [based on BRT models. 38](#_Toc211020217)

[Figure S3: Effects of major predictors (RCs >3%) for presence of](#_Toc211020218) *[Rhipicephalus microplus](#_Toc211020218)* [based on BRT models. 39](#_Toc211020218)

[Figure S4: The recorded and predicted distributions of](#_Toc211020219) *[Dermacentor reticulatus](#_Toc211020219)* [in Europe. 40](#_Toc211020219)

[Figure S5: The recorded and predicted distributions of](#_Toc211020220) *[Rhipicephalus microplus](#_Toc211020220)* [within global range. 41](#_Toc211020220)

[Figure S6: Predictive performance of the three machine-learning algorithms. 42](#_Toc211020221)

[Figure S7: Effects of major predictors (RCs >5%) for presence of](#_Toc211020222) *[Babesia microti](#_Toc211020222)* [based on RF models. 44](#_Toc211020222)

[Figure S8: Effects of major predictors (RCs >5%) for presence of](#_Toc211020223) *[Babesia](#_Toc211020223)* [sp. venatorum based on RF models. 45](#_Toc211020223)

[Figure S9: Effects of major predictors (RCs >5%) for presence of](#_Toc211020224) *[Babesia divergens](#_Toc211020224)* [based on RF models. 46](#_Toc211020224)

[Figure S10: Effects of major predictors (RCs >5%) for presence of](#_Toc211020225) *[Babesia bigemina](#_Toc211020225)* [based on RF models. 47](#_Toc211020225)

[Figure S11: Effects of major predictors (RCs >5%) for presence of](#_Toc211020226) *[Babesia bovis](#_Toc211020226)* [based on RF models. 48](#_Toc211020226)

[Figure S12: Effects of major predictors (RCs >5%) for presence of](#_Toc211020227) *[Babesia odocoilei](#_Toc211020227)* [based on RF models. 49](#_Toc211020227)

[Figure S13: The recorded and predicted distributions of](#_Toc211020228) *[Babesia microti](#_Toc211020228)* [within global range. 50](#_Toc211020228)

[Figure S14: The recorded and predicted distributions of](#_Toc211020229) *[Babesia](#_Toc211020229)* [sp. venatorum in Eurasia. 51](#_Toc211020229)

[Figure S15: The recorded and predicted distributions of](#_Toc211020230) *[Babesia divergens](#_Toc211020230)* [in Eurasia and Africa. 52](#_Toc211020230)

[Figure S16: The recorded and predicted distributions of](#_Toc211020231) *[Babesia bigemina](#_Toc211020231)* [within global range. 53](#_Toc211020231)

[Figure S17: The recorded and predicted distributions of](#_Toc211020232) *[Babesia bovis](#_Toc211020232)* [within global range. 54](#_Toc211020232)

[Figure S18: The recorded and predicted distributions of](#_Toc211020233) *[Babesia odocoilei](#_Toc211020233)* [in Americas. 55](#_Toc211020233)

#

# Table S1: The inclusion and exclusion criteria of screening publications.

| **Criteria** | **Guidance** | **Outcome** |
| --- | --- | --- |
| **Title/Abstract screening** |  |  |
| #1: Disease | Does the Title/Abstract refer the pathogens which belong to *Babesia*? | If Yes, remain and evaluate #2. If No, exclude. |
| #2: Pathogens | Does the Title/Abstract refer the pathogens which are detected from natural environment? | If Yes, remain and evaluate #3. If No, exclude. |
| #3: Tested objects | Does the Title/Abstract refer the pathogens which are detected from vectors, animals, or humans? | If Yes, remain and evaluate #4. If No, exclude. |
| #4: Not review | Does the Title/Abstract refer the article which is Not a review? (Not presenting new primary data) | If No, remain for full text review. If Yes, exclude. |
| **Full text screening** |  |  |
| #1: Re-screening | Does the article meet the screening criteria following? 1-pathogens belong to *Babesia* 2-infection in natural environment 3-detected from vectors, animals, or humans 4-not drug or vaccine trials without geographical or clinical information of cases 5-not research of transmission mechanism in vectors and animals 6-not molecular or cellular structure and function research of *Babesia*  7-full-text availability in languages other than English | If Yes, remain and evaluate #2. If No, exclude. |
| #2: Laboratory method | Does the article refer the specific detection methods? 1-detailed specimen used for testing (vectors or blood from animals or humans) 2- testing method for pathogens (microscopical or molecular or pathogen isolation) 3- pathogen identified in the detection | If Yes, remain and evaluate #3. If No, exclude. |
| #3: Geographical information | Does the article provide the geographical information? 1-geographic location information at administrative division levels 2-exact locations or marked the latitude and longitude 3-explicit locations of getting infections or sampling | If Yes, remain for data set.  If No, exclude. |

# Table S2: The laboratory tests used to detect *Babesia* infections in the reviewed studies.

|  | **Detection methods** |
| --- | --- |
| **Infection in vectors** | (1) molecular detection and sequence determination;  (2) isolation and cultivation of pathogens from samples;  (3) light or electronic micrograph identification. |
| **Infection in animals** | 1. molecular detection with PCR or sequencing; 2. isolation and cultivation of pathogens from samples; 3. light or electronic micrograph identification; |
| **Confirmed human cases** | (1) molecular detection and sequence determination;  (2) isolation and cultivation of pathogens from samples;  (3) light or electronic micrograph identification. |

# Table S3: List of variables extracted from reviewed studies

| **Variables** | **Explanation** |
| --- | --- |
| Reference ID | Unique identifier assigned to an article. |
| Article title | Article title that included in the review. |
| Authors | Authors of the included article. |
| Publication year | Publication year of the article eligible for inclusion. |
| Study period | The start and end time for the period over which the sample collected. |
| Study site | Sampling sites including three parts to be recorded, country, the detailed address in the article and coordinates. Coordinates of geographic center of the detailed locations was used if they are not provided by author. |
| Detection method | The technology used to detect specific pathogen. |
| Pathogen name | Standard name of *Babesia* tested in the study. |
| Pathogen detected from vectors/animals |  |
| Species of vectors/animals | The species of detected vectors/animals. |
| Number of tested all | The total number tested for specific pathogen. |
| Number of tested positive | The number tested as positive for specific pathogen. |
| Pathogen detected from human beings |  |
| Number of individuals tested all | The number of individuals tested all for specific pathogen. |
| Number of individuals tested positive | The number of individuals tested as positive for specific pathogen. |
| Clinical manifestation | Record the clinical manifestation of humans if they were only infected by one species of pathogen and infection type was confirmed infection. |

# Table S4: The references for all the *Babesia* species.

| ***Babesia* species** | **N** | **Reference ID** |
| --- | --- | --- |
| ***Babesia microti*** | **349** | **1, 2, 3, 5, 6, 7, 11, 12, 15, 19, 23, 25, 26, 30, 67, 68, 69, 70, 71, 72, 73, 74, 75, 76, 80, 81, 87, 89, 91, 95, 96, 102, 106, 107, 108, 110, 111, 118, 122, 123, 124, 125, 126, 127, 128, 129, 130, 131, 132, 133, 135, 136, 137, 138, 139, 140, 141, 142, 144, 156, 157, 159, 167, 168, 171, 174, 175, 176, 177, 180, 183, 185, 186, 188, 190, 191, 192, 193, 195, 198, 199, 202, 205, 206, 209, 210, 211, 212, 213, 215, 218, 219, 220, 221, 232, 233, 236, 251, 269, 270, 280, 282, 285, 286, 287, 289, 292, 293, 301, 304, 305, 306, 320, 321, 322, 323, 332, 333, 334, 335, 336, 340, 341, 342, 344, 345, 348, 349, 353, 354, 377, 380, 381, 385, 387, 388, 392, 396, 398, 399, 400, 407, 409, 439, 445, 450, 452, 453, 457, 461, 462, 466, 469, 472, 475, 476, 484, 485, 486, 490, 491, 492, 496, 497, 498, 499, 500, 501, 502, 503, 504, 505, 517, 519, 525, 529, 531, 535, 536, 541, 542, 546, 547, 554, 555, 556, 560, 562, 563, 566, 570, 572, 611, 620, 624, 629, 635, 639, 643, 657, 659, 660, 663, 671, 681, 687, 691, 697, 702, 712, 729, 730, 731, 735, 745, 754, 755, 757, 759, 766, 770, 784, 797, 798, 799, 800, 801, 802, 803, 804, 805, 817, 818, 823, 827, 828, 829, 833, 837, 840, 848, 852, 853, 854, 855, 862, 863, 865, 866, 867, 868, 869, 870, 871, 881, 882, 885, 888, 890, 891, 894, 898, 899, 900, 901, 903, 907, 910, 912, 914, 916, 925, 926, 927, 928, 931, 933, 936, 938, 941, 945, 949, 957, 963, 964, 965, 972, 973, 974, 977, 978, 980, 982, 983, 984, 985, 992, 993, 997, 999, 1006, 1011, 1013, 1015, 207, 312, 329, 339, 404, 406, 527, 689, 690, 779, 809, 825, 826, 886, 906, 913, 934, 1062, 1018, 1029, 1031, 1033, 1034, 1036, 1037, 1039, 1047, 1048, 1049, 1050, 1051, 1053, 1054, 1057, 1058, 1059, 1060, 1064, 1065, 1067, 1068, 1075, 1078, 1081, 820** |
| ***Babesia sp. venatorum*** | **132** | **4, 9, 10, 13, 15, 63, 84, 85, 87, 89, 91, 92, 94, 95, 96, 98, 157, 183, 186, 190, 196, 197, 209, 233, 250, 266, 274, 278, 282, 293, 297, 299, 301, 302, 316, 318, 320, 321, 324, 330, 331, 332, 336, 351, 370, 389, 402, 417, 424, 439, 466, 469, 472, 493, 507, 517, 519, 520, 543, 546, 547, 550, 556, 558, 562, 563, 564, 594, 611, 620, 633, 639, 643, 646, 659, 696, 734, 735, 746, 748, 753, 756, 757, 777, 778, 780, 781, 804, 805, 818, 821, 829, 834, 836, 837, 838, 853, 855, 858, 865, 885, 894, 899, 901, 902, 903, 905, 918, 919, 936, 968, 977, 1012, 1015, 50, 181, 412, 528, 832, 1018, 1027, 1031, 1032, 1033, 1037, 1039, 1043, 1045, 1066, 1074, 1079, 1080** |
| ***unknown*** | **123** | **10, 15, 16, 83, 86, 89, 90, 103, 105, 114, 115, 117, 120, 145, 147, 155, 157, 169, 179, 196, 214, 225, 231, 232, 233, 238, 241, 246, 247, 248, 252, 254, 255, 265, 268, 277, 282, 303, 309, 310, 311, 316, 319, 343, 346, 356, 371, 382, 383, 386, 391, 412, 415, 418, 421, 424, 439, 456, 458, 474, 477, 479, 493, 506, 510, 513, 533, 540, 543, 545, 547, 553, 561, 565, 593, 611, 625, 630, 661, 664, 669, 674, 706, 710, 718, 719, 724, 725, 733, 735, 741, 760, 761, 765, 768, 772, 775, 783, 792, 793, 803, 831, 845, 847, 861, 874, 876, 877, 880, 893, 904, 909, 922, 932, 936, 937, 988, 989, 990, 998, 1001, 1066, 1079** |
| ***Babesia* species** | **N** | **Reference ID** |
| ***Babesia canis*** | **118** | **8, 21, 27, 28, 39, 40, 41, 42, 43, 44, 54, 58, 59, 64, 83, 95, 102, 121, 162, 163, 164, 178, 184, 186, 187, 204, 208, 210, 216, 226, 243, 250, 262, 263, 282, 290, 291, 310, 313, 314, 316, 331, 332, 337, 355, 357, 366, 379, 390, 401, 405, 425, 430, 468, 475, 489, 519, 522, 537, 547, 549, 556, 590, 597, 606, 608, 609, 614, 626, 632, 637, 666, 685, 693, 700, 718, 727, 742, 743, 758, 771, 775, 787, 789, 794, 806, 811, 812, 813, 837, 859, 860, 865, 872, 881, 901, 918, 939, 958, 967, 970, 979, 995, 1014, 315, 412, 539, 844, 934, 975, 1001, 1022, 1028, 1037, 1056, 1061, 1069, 1071** |
| ***Babesia bigemina*** | **115** | **18, 35, 36, 109, 116, 134, 146, 149, 152, 154, 223, 230, 257, 258, 283, 284, 289, 294, 295, 310, 313, 327, 359, 364, 365, 372, 373, 374, 375, 376, 378, 411, 423, 428, 438, 446, 458, 466, 481, 483, 487, 488, 519, 526, 559, 567, 568, 576, 578, 580, 584, 585, 587, 591, 595, 600, 601, 603, 604, 607, 610, 616, 619, 631, 645, 647, 648, 658, 670, 672, 675, 678, 706, 709, 715, 716, 717, 721, 722, 723, 726, 728, 733, 738, 739, 758, 775, 786, 787, 790, 795, 808, 816, 821, 836, 841, 843, 849, 856, 878, 879, 883, 895, 911, 915, 950, 954, 955, 959, 987, 1007, 1016, 1022, 1024, 1063** |
| ***Babesia divergens*** | **98** | **9, 13, 15, 30, 50, 51, 52, 53, 87, 88, 89, 92, 94, 95, 104, 120, 153, 173, 183, 192, 193, 196, 201, 203, 233, 250, 264, 284, 290, 295, 313, 323, 324, 332, 351, 358, 361, 393, 394, 395, 397, 411, 422, 448, 466, 508, 517, 519, 530, 543, 546, 573, 602, 605, 611, 634, 639, 659, 696, 702, 706, 749, 763, 780, 801, 802, 818, 829, 834, 837, 838, 855, 857, 858, 865, 882, 885, 889, 894, 897, 903, 935, 941, 960, 961, 962, 966, 969, 981, 991, 826, 842, 1022, 1032, 1039, 1074, 1079, 1080,** |
| ***Babesia vogeli*** | **98** | **14, 17, 40, 45, 59, 93, 97, 101, 112, 143, 159, 160, 162, 163, 165, 166, 189, 217, 216, 222, 239, 240, 244, 245, 247, 275, 279, 282, 288, 290, 310, 314, 316, 326, 332, 363, 420, 427, 432, 434, 435, 436, 449, 455, 480, 523, 557, 571, 581, 583, 586, 608, 609, 640, 641, 642, 644, 652, 654, 656, 662, 667, 668, 669, 676, 679, 688, 692, 693, 703, 710, 714, 736, 737, 742, 744, 752, 788, 789, 791, 807, 813, 819, 846, 873, 875, 953, 976, 1001, 1002, 1029, 1030, 1038, 1040, 1042, 1070, 1072, 1073** |
| ***Babesia bovis*** | **88** | **36, 37, 109, 116, 134, 146, 152, 154, 223, 230,259, 276, 283, 284, 290, 294, 310, 313, 364, 372, 373, 374, 375, 378, 423, 428, 447, 458, 481, 487, 509, 519, 526, 534, 559, 576, 577, 578, 580, 584, 585, 587, 600, 603, 604, 607, 610, 616, 617, 619, 628, 631, 670, 672, 677, 709, 722, 723, 728, 732, 733, 738, 739, 740, 775, 785, 786, 787, 808, 816, 841, 849, 856, 865, 893, 895, 911, 943, 950, 987, 426, 1021, 1022, 1024, 1025, 1041, 1063, 1076** |
| ***Babesia caballi*** | **59** | **20, 38, 159, 183, 237, 260, 261, 277, 296, 298, 310, 313, 326, 368, 437, 460, 544, 548, 550, 574, 582, 589, 595, 599, 622, 623, 649, 653, 654, 655, 705, 707, 708, 711, 721, 771, 775, 796, 811, 835, 847, 851, 865, 896, 915, 917, 946, 947, 948, 951, 952, 956, 986, 1008, 329, 429, 884, 1019, 1020** |
| ***Babesia* species** | **N** | **Reference ID** |
| ***Babesia gibsoni*** | **53** | **55, 56, 57, 58, 59, 60, 61, 64, 158, 159, 161, 164, 187, 222, 310, 317, 362, 363, 367, 408, 431, 432, 435, 440, 449, 465, 473, 519, 586, 612, 615, 652, 667, 668, 673, 692, 699, 703, 710, 712, 713, 727, 807, 811, 813, 858, 958, 1009, 538, 814, 1002, 1029, 1030** |
| ***Babesia capreoli*** | **45** | **9, 10, 13, 46, 88, 89, 94, 190, 196, 301, 316, 320, 324, 336, 347, 424, 489, 513, 524, 556, 562, 563, 633, 639, 696, 734, 735, 748, 751, 780, 804, 821, 834, 836, 837, 838, 853, 854, 865, 885, 419, 1018, 1031, 1037, 1079** |
| ***Babesia ovis*** | **36** | **102, 144, 200, 224, 230, 307, 308, 360, 369, 414, 433, 441, 468, 512, 514, 518, 549, 575, 613, 650, 651, 686, 695, 704, 815, 830, 850, 887, 892, 937, 1004, 1010, 1026, 1044, 1046, 1080** |
| ***Babesia odocoilei*** | **25** | **82, 96, 199, 251, 254, 255, 271, 272, 273, 322, 323, 352, 519, 536, 570, 577, 636, 720, 735, 834, 994, 996, 1003, 1005, 1082** |
| ***Babesia occultans*** | **21** | **144, 210, 277, 413, 438, 451, 471, 511, 519, 522, 597, 681, 684, 847, 915, 1007, 1016, 1019, 1025, 1044, 1056** |
| ***Babesia cf. microti*** | **18** | **22, 62, 77, 78, 79, 113, 150, 253, 454, 459, 495, 743, 776, 864, 920, 1000, 1043, 1080** |
| ***Babesia motasi*** | **15** | **94, 149, 230, 235, 277, 281, 325, 360, 369, 438, 518, 618, 721, 944, 971** |
| ***Babesia vulpes*** | **13** | **170, 356, 425, 597, 638, 736, 750, 751, 764, 782, 837, 905, 923** |
| ***Babesia cf. divergens*** | **9** | **88, 251, 469, 472, 519, 698, 1065, 1079, 1080** |
| ***Babesia major*** | **9** | **92, 277, 281, 442, 517, 522, 648, 937, 1016** |
| ***Babesia crassa*** | **8** | **49, 210, 325, 331, 332, 522, 597, 1056** |
| ***Babesia duncani*** | **8** | **344, 444,494, 551, 696, 773, 1017, 1052** |
| ***Babesia cf. odocoilei*** | **5** | **9, 10, 780, 419, 1035** |
| ***Babesia lengau*** | **5** | **65, 66, 470, 822, 839** |
| ***Babesia vesperuginis*** | **5** | **99, 100, 297, 331, 598** |
| ***Babesia felis*** | **4** | **519, 657, 770, 839** |
| ***Babesia leo*** | **4** | **656, 657, 770,839** |
| ***Babesia ovata*** | **4** | **373, 519, 810 733** |
| ***Babesia sp. badger type A*** | **4** | **356, 482, 638, 751** |
| ***Babesia annae*** | **3** | **29, 350 735,** |
| ***Babesia conradae*** | **3** | **47, 48, 338** |
| ***Babesia naoakii*** | **3** | **672, 738, 739** |
| ***Babesia sp. AJB-2006*** | **3** | **249, 866, 888** |
| ***Babesia sp. Anglona/AA-2011*** | **3** | **194, 680, 747** |
| ***Babesia sp. UR1*** | **3** | **519, 629, 694** |
| ***Babesia sp. Xinjiang*** | **3** | **464, 518, 921** |
| ***Babesia sp. tavsan1*** | **3** | **210, 597, 1056** |
| ***Babesia sp. tavsan2*** | **3** | **521, 597, 1056** |
| ***Babesia bicornis*** | **2** | **33, 34** |
| ***Babesia cf. crassa*** | **2** | **328, 665** |
| ***Babesia* species** | **N** | **Reference ID** |
| ***Babesia hongkongensis*** | **2** | **267, 693** |
| ***Babesia negevi*** | **2** | **769, 1040** |
| ***Babesia orientalis*** | **2** | **289, 942** |
| ***Babesia pecorum*** | **2** | **552, 1020** |
| ***Babesia sp. 28*** | **2** | **515, 519** |
| ***Babesia sp. 4 NAN-2012*** | **2** | **519, 638** |
| ***Babesia sp. Bime*** | **2** | **466, 493** |
| ***Babesia sp. Coco*** | **2** | **888, 1055** |
| ***Babesia sp. Irk-Hc133*** | **2** | **325, 472** |
| ***Babesia sp. Iwate248*** | **2** | **410, 416** |
| ***Babesia sp. Kh-Hc222*** | **2** | **325, 472** |
| ***Babesia sp. Kh-Hj143*** | **2** | **472, 519** |
| ***Babesia sp. NV-1*** | **2** | **519, 592** |
| ***Babesia sp. OO-2012*** | **2** | **10, 838** |
| ***Babesia sp. SAP#091*** | **2** | **519, 683** |
| ***Babesia sp. Ucbas*** | **2** | **210, 1056** |
| ***Babesia sp. YZ-2012*** | **2** | **466, 519** |
| ***Babesia sp. d79*** | **2** | **92, 94** |
| ***Babesia sp. deer clade*** | **2** | **300, 324** |
| ***Babesia sp. giraffe 1505*** | **2** | **516, 519** |
| ***Babesia sp. sable antelope/2005*** | **2** | **511, 519** |
| ***Babesia ardeae*** | **1** | **924** |
| ***Babesia behnkei*** | **1** | **31** |
| ***Babesia bennetti*** | **1** | **32** |
| ***Babesia cf. lengau ex Hyaenidae JK-2021*** | **1** | **470** |
| ***Babesia cf. motasi*** | **1** | **467** |
| ***Babesia cf. vogeli ex Crocuta crocuta JK-2021*** | **1** | **470** |
| ***Babesia kiwiensis*** | **1** | **596** |
| ***Babesia lohae*** | **1** | **569** |
| ***Babesia mackerrasorum*** | **1** | **569** |
| ***Babesia panickeri*** | **1** | **701** |
| ***Babesia peircei*** | **1** | **783** |
| ***Babesia pisicii*** | **1** | **83** |
| ***Babesia poelea*** | **1** | **227** |
| ***Babesia sp. 1 1093 cl9*** | **1** | **995** |
| ***Babesia sp. 1 JM-2013*** | **1** | **148** |
| ***Babesia sp. 1 JY-2013*** | **1** | **682** |
| ***Babesia sp. 1 ZH-2016*** | **1** | **96** |
| ***Babesia* species** | **N** | **Reference ID** |
| ***Babesia sp. 10*** | **1** | **515** |
| ***Babesia sp. 10 1092 cl9*** | **1** | **995** |
| ***Babesia sp. 11 1095*** | **1** | **995** |
| ***Babesia sp. 12 1101*** | **1** | **995** |
| ***Babesia sp. 16*** | **1** | **515** |
| ***Babesia sp. 18*** | **1** | **515** |
| ***Babesia sp. 2 1092 cl4*** | **1** | **995** |
| ***Babesia sp. 2 BCS-2013*** | **1** | **839** |
| ***Babesia sp. 25*** | **1** | **1008** |
| ***Babesia sp. 3*** | **1** | **1008** |
| ***Babesia sp. 3 1093 cl8*** | **1** | **995** |
| ***Babesia sp. 33*** | **1** | **515** |
| ***Babesia sp. 4*** | **1** | **1008** |
| ***Babesia sp. 4 1093 cl2*** | **1** | **995** |
| ***Babesia sp. 49*** | **1** | **515** |
| ***Babesia sp. 5 1093 cl7*** | **1** | **995** |
| ***Babesia sp. 51*** | **1** | **515** |
| ***Babesia sp. 6 1092 cl1*** | **1** | **995** |
| ***Babesia sp. 6ME22A*** | **1** | **781** |
| ***Babesia sp. 7 1092 cl3*** | **1** | **995** |
| ***Babesia sp. 8 1092 cl5*** | **1** | **995** |
| ***Babesia sp. 9 1093 cl1*** | **1** | **995** |
| ***Babesia sp. A360_1-AP2017*** | **1** | **1023** |
| ***Babesia sp. AKT7*** | **1** | **673** |
| ***Babesia sp. ALT-2012*** | **1** | **774** |
| ***Babesia sp. AR1*** | **1** | **352** |
| ***Babesia sp. AZ*** | **1** | **384** |
| ***Babesia sp. AdN3*** | **1** | **88** |
| ***Babesia sp. Akita610*** | **1** | **363** |
| ***Babesia sp. Akita615*** | **1** | **363** |
| ***Babesia sp. Am-Hc344*** | **1** | **472** |
| ***Babesia sp. Ankara*** | **1** | **627** |
| ***Babesia sp. BCS-2013a*** | **1** | **327** |
| ***Babesia sp. BCS-2013b*** | **1** | **327** |
| ***Babesia sp. BCS-2013c*** | **1** | **327** |
| ***Babesia sp. BCS-2013d*** | **1** | **327** |
| ***Babesia sp. BCS-2013e*** | **1** | **327** |
| ***Babesia sp. BCS-2013f*** | **1** | **327** |
| ***Babesia sp. BiCM002*** | **1** | **81** |
| ***Babesia* species** | **N** | **Reference ID** |
| ***Babesia sp. Brazilian pampas cat/MRA-2011*** | **1** | **579** |
| ***Babesia sp. C1 AAM-2008*** | **1** | **53** |
| ***Babesia sp. CA*** | **1** | **384** |
| ***Babesia sp. CA-02*** | **1** | **908** |
| ***Babesia sp. CA-03*** | **1** | **908** |
| ***Babesia sp. CA-04*** | **1** | **908** |
| ***Babesia sp. CA-05*** | **1** | **908** |
| ***Babesia sp. CA-06*** | **1** | **908** |
| ***Babesia sp. CO-01*** | **1** | **908** |
| ***Babesia sp. CS58*** | **1** | **648** |
| ***Babesia sp. CSP-2014*** | **1** | **929** |
| ***Babesia sp. California RD61*** | **1** | **930** |
| ***Babesia sp. China-BQ1*** | **1** | **24** |
| ***Babesia sp. CoatiCR*** | **1** | **1077** |
| ***Babesia sp. D1 AAM-2008*** | **1** | **53** |
| ***Babesia sp. D2 AAM-2008*** | **1** | **53** |
| ***Babesia sp. D3 AAM-2008*** | **1** | **53** |
| ***Babesia sp. DK208*** | **1** | **310** |
| ***Babesia sp. DMA-2015*** | **1** | **773** |
| ***Babesia sp. DO23163*** | **1** | **751** |
| ***Babesia sp. EM-2016*** | **1** | **443** |
| ***Babesia sp. F1*** | **1** | **119** |
| ***Babesia sp. F2*** | **1** | **119** |
| ***Babesia sp. F3*** | **1** | **119** |
| ***Babesia sp. F4*** | **1** | **119** |
| ***Babesia sp. FL-02*** | **1** | **908** |
| ***Babesia sp. FL-03*** | **1** | **908** |
| ***Babesia sp. FL-04*** | **1** | **908** |
| ***Babesia sp. FL-05*** | **1** | **908** |
| ***Babesia sp. FL-06*** | **1** | **908** |
| ***Babesia sp. FR1*** | **1** | **767** |
| ***Babesia sp. Fukui766*** | **1** | **363** |
| ***Babesia sp. G17M*** | **1** | **310** |
| ***Babesia sp. GA-04*** | **1** | **908** |
| ***Babesia sp. GA-05*** | **1** | **908** |
| ***Babesia sp. GA-06*** | **1** | **908** |
| ***Babesia sp. GoA3*** | **1** | **88** |
| ***Babesia sp. H10*** | **1** | **631** |
| ***Babesia sp. H4*** | **1** | **1004** |
| ***Babesia* species** | **N** | **Reference ID** |
| ***Babesia sp. HLJ-8*** | **1** | **466** |
| ***Babesia sp. Hebei*** | **1** | **467** |
| ***Babesia sp. Hebei-2005*** | **1** | **24** |
| ***Babesia sp. Hue-1*** | **1** | **463** |
| ***Babesia sp. Human KY*** | **1** | **532** |
| ***Babesia sp. I38*** | **1** | **242** |
| ***Babesia sp. ID-01*** | **1** | **908** |
| ***Babesia sp. IoRK/HM101*** | **1** | **1006** |
| ***Babesia sp. Irk-Hc129*** | **1** | **472** |
| ***Babesia sp. Irk-Hc130*** | **1** | **472** |
| ***Babesia sp. Irk-Ip256*** | **1** | **472** |
| ***Babesia sp. Irk-Ip279*** | **1** | **472** |
| ***Babesia sp. Irk-Ip525*** | **1** | **472** |
| ***Babesia sp. Irk-Ip655*** | **1** | **472** |
| ***Babesia sp. JL-2016a*** | **1** | **621** |
| ***Babesia sp. JPM-2014*** | **1** | **588** |
| ***Babesia sp. K-2015*** | **1** | **478** |
| ***Babesia sp. K4*** | **1** | **119** |
| ***Babesia sp. KMG-2009a*** | **1** | **234** |
| ***Babesia sp. KO1*** | **1** | **403** |
| ***Babesia sp. Kh-Hc232*** | **1** | **472** |
| ***Babesia sp. Kh-Hj131*** | **1** | **472** |
| ***Babesia sp. Kh-Hj394*** | **1** | **472** |
| ***Babesia sp. Kh-Hj42*** | **1** | **472** |
| ***Babesia sp. Kh-Hj441*** | **1** | **472** |
| ***Babesia sp. Kh-Hj540*** | **1** | **472** |
| ***Babesia sp. Liaoning-2005*** | **1** | **24** |
| ***Babesia sp. M1*** | **1** | **119** |
| ***Babesia sp. MA#230*** | **1** | **519** |
| ***Babesia sp. MA#361-1*** | **1** | **683** |
| ***Babesia sp. MA-2016a*** | **1** | **686** |
| ***Babesia sp. MA361-2*** | **1** | **519** |
| ***Babesia sp. MML-2014*** | **1** | **1000** |
| ***Babesia sp. MN-01*** | **1** | **908** |
| ***Babesia sp. MN-03*** | **1** | **908** |
| ***Babesia sp. MO-01*** | **1** | **908** |
| ***Babesia sp. MO-03*** | **1** | **908** |
| ***Babesia sp. MO-04*** | **1** | **908** |
| ***Babesia sp. MO-05*** | **1** | **908** |
| ***Babesia sp. MT61*** | **1** | **242** |
| ***Babesia* species** | **N** | **Reference ID** |
| ***Babesia sp. MT753*** | **1** | **242** |
| ***Babesia sp. MT763*** | **1** | **242** |
| ***Babesia sp. MT775*** | **1** | **242** |
| ***Babesia sp. Madang-2005*** | **1** | **24** |
| ***Babesia sp. MzN6*** | **1** | **88** |
| ***Babesia sp. NG-2012*** | **1** | **606** |
| ***Babesia sp. NJ5*** | **1** | **519** |
| ***Babesia sp. O109*** | **1** | **310** |
| ***Babesia sp. ONT-03*** | **1** | **908** |
| ***Babesia sp. OrkunO-2021a*** | **1** | **228** |
| ***Babesia sp. PA-01*** | **1** | **908** |
| ***Babesia sp. PA-02*** | **1** | **908** |
| ***Babesia sp. PA-03*** | **1** | **908** |
| ***Babesia sp. PA-04*** | **1** | **908** |
| ***Babesia sp. Punjab*** | **1** | **465** |
| ***Babesia sp. RETV-2015*** | **1** | **371** |
| ***Babesia sp. RWF-2013*** | **1** | **519** |
| ***Babesia sp. Rabbit 774 Nantucket*** | **1** | **532** |
| ***Babesia sp. Rabbit 831 Nantucket*** | **1** | **532** |
| ***Babesia sp. SAP#131*** | **1** | **519** |
| ***Babesia sp. SH-2015a*** | **1** | **325** |
| ***Babesia sp. Suis*** | **1** | **824** |
| ***Babesia sp. TVY 11.016*** | **1** | **151** |
| ***Babesia sp. TVY 12.047*** | **1** | **151** |
| ***Babesia sp. TVY 14.010*** | **1** | **151** |
| ***Babesia sp. TVY 14.019*** | **1** | **151** |
| ***Babesia sp. TVY 14.022*** | **1** | **151** |
| ***Babesia sp. TVY 9.003*** | **1** | **151** |
| ***Babesia sp. TX-01*** | **1** | **908** |
| ***Babesia sp. Tianzhu-2005*** | **1** | **24** |
| ***Babesia sp. UR2*** | **1** | **629** |
| ***Babesia sp. WV-01*** | **1** | **908** |
| ***Babesia sp. WV-02*** | **1** | **908** |
| ***Babesia sp. WV-04*** | **1** | **908** |
| ***Babesia sp. Western Cape*** | **1** | **770** |
| ***Babesia sp. XXB/HangZhou*** | **1** | **172** |
| ***Babesia sp. Xinjiang-2005*** | **1** | **24** |
| ***Babesia sp. YLG*** | **1** | **182** |
| ***Babesia* species** | **N** | **Reference ID** |
| ***Babesia sp. badger*** | **1** | **736** |
| ***Babesia sp. badger type B*** | **1** | **751** |
| ***Babesia sp. capybara 1*** | **1** | **773** |
| ***Babesia sp. d6*** | **1** | **92** |
| ***Babesia sp. ex Cervus nippon*** | **1** | **969** |
| ***Babesia sp. genet/MRA-2011*** | **1** | **579** |
| ***Babesia sp. giraffe 0105*** | **1** | **516** |
| ***Babesia sp. giraffe 229*** | **1** | **516** |
| ***Babesia sp. giraffe 544*** | **1** | **516** |
| ***Babesia sp. maned wolf*** | **1** | **866** |
| ***Babesia sp. pudui*** | **1** | **940** |
| ***Babesia sp. red deer/G1/IRL*** | **1** | **104** |
| ***Babesia sp. spanish dog*** | **1** | **159** |
| ***Babesia sp. strain TXCTR2*** | **1** | **256** |
| ***Babesia sp. strain TXCTR3*** | **1** | **256** |
| ***Babesia ugwidiensis*** | **1** | **783** |
| ***Babesia uriae*** | **1** | **229** |
| ***Babesia vitalii*** | **1** | **762** |

# Table S5: The spatial resolution, study duration and source of the included data.

| **Variable** | **Spatial resolution** | **Study duration** | **Source of data** | **Website** | **Reference** |
| --- | --- | --- | --- | --- | --- |
| Climate data | 0°2·5′ | 1975‒2019 | WorldClim | https://www.worldclim.org/ | Fick SE, Hijmans RJ. WorldClim 2: new 1-km spatial resolution climate surfaces for global land areas. Int. J. Climatol., 2017; 37: 4302-15.  Harris I, Jones PD, Osborn TJ, Lister DH. Updated high-resolution grids of monthly climatic observations – the CRU TS3.10 Dataset. Int. J. Climatol., 2014; 34: 623-42. |
| Leaf area index | 8km | 1981‒2019 | Resource and Environment Science and Data Center | https://www.resdc.cn/ | Yang L, Liu R, Chen JM. Retrospective retrieval of long-term consistent global leaf area index (1981-2011) from combined AVHRR and MODIS data. J Geophys Res Biogeosci, 2015; 117. |
| Land cover | 0·3km | 1992‒2019 | European Space Agency | https://maps.elie.ucl.ac.be/CCI/ | European Space Agency. ESA Land Cover Climate Change Initiative (Land_Cover_cci): Global Land Cover Maps, Version 2.0.7. https://catalogue.ceda.ac.uk/uuid/b382ebe6679d44b8b0e68ea4ef4b701c/ (accessed May 28, 2021) |
| Human Footprint | 1km | 2000‒2018 | Scientific Data | https://www.gisrsdata.com/ | Mu, Haowei; Li, Xuecao; Wen, Yanan; Huang, Jianxi; Du, Peijun; Su, Wei; et al. (2021): An annual global terrestrial Human Footprint dataset from 2000 to 2018. figshare. Figure. https://doi.org/10.6084/m9.figshare.16571064.v5 |
| Elevation | 1km | 2010 | EarthEnv (DEM90) | http://www.earthenv.org/ | Robinson N, Regetz J, Guralnick RP. EarthEnv-DEM90: A nearly-global, void-free, multi-scale smoothed, 90m digital elevation model from fused ASTER and SRTM data. ISPRS, 2014; 87: 57-67. |
| Livestock density | 1km | 2010; 2015 | Food and Agriculture Organization (FAO) | http://www.fao.org/livestock-systems/en/ | Gilbert M, Nicolas G, Cinardi G, et al. Global distribution data for cattle, buffaloes, horses, sheep, goats, pigs, chickens and ducks in 2010. Sci Data, 2018; 5: 180227. |
| Mammalian richness | 0°0′30″ | 2013 | International Union for Conservation of Nature (IUCN) | https://sedac.ciesin.columbia.edu/ | International Union for Conservation of Nature - IUCN, and Center for International Earth Science Information Network - CIESIN - Columbia University. 2015. Gridded Species Distribution: Global Mammal Richness Grids, 2015 Release. Palisades, NY: NASA Socioeconomic Data and Applications Center (SEDAC). |
| Rodent richness | 10km | 2018 | BiodiversityMapping.org | https://biodiversitymapping.org/index.php/permissions/ | Jenkins, C.N. & K. Van Houtan. (2016). Global and regional priorities for marine biodiversity protection. Pimm, SL, CN Jenkins, R Abell, TM Brooks, JL Gittleman, LN Joppa, PH Raven, CM Roberts, JO Sexton (2014) The biodiversity of species and their rates of extinction, distribution, and protection. Science 344(6187): 1246752. |
| Population number | 1km | 2020 | WorldPop 2020 | https://www.worldpop.org/ | WorldPop. Population counts, unconstrained global mosaics 2000-2020 (1 km resolution), 2020. https://www.worldpop.org/geodata/listing?id=64/ (accessed Apr 12, 2021). |
| Global downscaled GDP | 0°2·5′ | 1990, 2025 | NASA Socioeconomic Data and Applications Center (SEDAC) | https://sedac.ciesin.columbia.edu/ | Gaffin SR, Xing X, Yetman G. Global 15 x 15 Minute Grids of the Downscaled GDP Based on the SRES B2 Scenario, 1990 and 2025. Palisades, NY: NASA Socioeconomic Data and Applications Center (SEDAC); 2004. |

# Table S6: Ecological factors potentially associated with the ticks and tick pathogens used in the modelling analysis.

| **Variable** | **Reference** | **Content** | **Method** | **Usage and/or result** |
| --- | --- | --- | --- | --- |
| BIO1‒19 | Alkishe AA., et al. [1] | Tick in Europe | Model: MaxEnt | Used in the tick ecological niche model. |
|  | Zhang L, et.al. [2] | Tick in America | Model: MaxEnt | Precipitation of the driest month contributed 19.6% in the model. |
|  | Eisen RJ, et al. [3] | Tick in America | Model: Logistic regression, RF and MaxEnt | Precipitation of coldest quarter contributed 37.8‒70.3 in the model. |
|  | Wang SS, et.al. [4] | Tick in global scale | Model: MaxEnt | Used in the tick ecological niche model. |
| Leaf area index | Fuller T, et.al. [5] | Monkeypox disease in the Congo Basin | Model: Logistic regression and MaxEnt | Used in the monkeypox ecological niche model. |
|  | Zhang YY, et.al. [6] | Tick-borne pathogens in global scale | Model: Logistic regression and RF | Used in tick-borne pathogens ecological niche model. |
| Elevation | Miao D, et.al. [7] | Tick and tick-borne pathogens in China | Model: Logistic regression | Used in tick ecological niche model. |
| Mixed cropland and nature vegetation | Allen T, et.al. [8] | Emerging zoonotic diseases in global scale | Model: Logistic regression | Land cover was used in the analysis of zoonotic pathogens ecological niche model. |
| Shrubland | Allen T, et.al. [8] | Emerging zoonotic diseases in global scale | Model: Logistic regression | Land cover was used in the analysis of zoonotic pathogens ecological niche model. |
| Mixed tree, shrub and herbaceous | Allen T, et.al. [8] | Emerging zoonotic diseases in global scale | Model: Logistic regression | Land cover was used in the analysis of zoonotic pathogens ecological niche model. |
| Grassland | Allen T, et.al. [8] | Emerging zoonotic diseases in global scale | Model: Logistic regression | Land cover was used in the analysis of zoonotic pathogens ecological niche model. |
| Sparse vegetation land | Allen T, et.al. [8] | Emerging zoonotic diseases in global scale | Model: Logistic regression | Land cover was used in the analysis of zoonotic pathogens ecological niche model. |
| Flooded vegetation | Allen T, et.al. [8] | Emerging zoonotic diseases in global scale | Model: Logistic regression | Land cover was used in the analysis of zoonotic pathogens ecological niche model. |
| Bare areas | Allen T, et.al. [8] | Emerging zoonotic diseases in global scale | Model: Logistic regression | Land cover was used in the analysis of zoonotic pathogens ecological niche model. |
| Water body | Allen T, et.al. [8] | Emerging zoonotic diseases in global scale | Model: Logistic regression | Land cover was used in the analysis of zoonotic pathogens ecological niche model. |
| Cropland | Allen T, et.al. [8] | Emerging zoonotic diseases in global scale | Model: Logistic regression | Land cover was used in the analysis of zoonotic pathogens ecological niche model. |
| Urban construction land | Allen T, et.al. [8] | Emerging zoonotic diseases in global scale | Model: Logistic regression | Land cover was used in the analysis of zoonotic pathogens ecological niche model. |
| Population count | Miao D, et.al. [7] | Tick and tick-borne pathogens in China | Model: Logistic regression | Contributed 19.7% in the model |
|  | Allen T, et.al. [8] | Emerging zoonotic diseases in global scale | Model: Logistic regression | Used in the pathogens ecological niche model. |
|  | Jones KE, et al. [9] | Emerging disease in global scale | Model: Logistic regression | Used in the pathogens ecological niche model. |
| Global Downscaled GDP | Miao D, et.al. [10] | Tick and tick-borne pathogens in China | Model: Logistic regression | Used in tick ecological niche model. |
|  | Magalhães AR, et.al. [11] | Natural focal disease in global scale | Model: MaxEnt | The most significant predictive variables for relative variable importance = 37 ± 13% standard error. |
| Human Footprint | Gallardo B, et.al. [12] | The global distribution of invaders | Model: MaxEnt | Factors related to the human footprint explained a substantial amount (23% on average) of species distributions. |
|  | Skinner EB, et.al. [13] | The vector-borne diseases | Model：RF | Human footprint is an important predictor of local occurrence and that its nonlinear effects vary predictably with the transmission ecology of each vector-borne diseases |
| Rodentia richness | Usman S, et.al. [14] | Transmission dynamic of MPXV | Model: SEIR | Used in the monkeypox dynamic model. |
| Mammalian richness | Olival KJ, et.al. [15] | Zoonotic spillover from mammals | Model: GAM | Used in the spillover model. |

1. Alkishe AA, Peterson AT, Samy AM. Climate change influences on the potential geographic distribution of the disease vector tick *Ixodes ricinus*. *PLoS One*. 2017; 12: e0189092.
2. Zhang L, Ma D, Li C, Zhou R, Wang J, Liu Q. Projecting the potential distribution areas of *Ixodes scapularis* (Acari: Ixodidae) driven by climate change. *Biology*. 2022; 11:107.
3. Eisen RJ, Feirer S, Padgett KA, et al. Modeling climate suitability of the western blacklegged tick in California. *J Med Entomol*. 2018; 55: 1133-42.
4. Wang SS, Liu JY, Wang BY, et al. Geographical distribution of Ixodes persulcatus and associated pathogens: Analysis of integrated data from a China field survey and global published data. *One Health*. 2023; 16: 100508.
5. Fuller T, Thomassen HA, Mulembakani PM, et al. Using remote sensing to map the risk of human monkeypox virus in the Congo Basin. *Ecohealth*. 2011; **8:** 14-25.
6. Zhang YY, Liu W, Fang LQ, et al. Mapping the global distribution of spotted fever group rickettsiae: a systematic review with modelling analysis. Lancet Digit Health. 2023; 5: e5-e15.
7. Miao D, Liu W, Fang LQ, et al. Mapping the global potential transmission hotspots for severe fever with thrombocytopenia syndrome by machine learning methods. *Emerg Microbes Infect.* 2020; **9:** 817-26.
8. Allen T, Murray KA, Zambrana-Torrelio C, et al. Global hotspots and correlates of emerging zoonotic diseases. *Nat Commun*. 2017; **8:** 1124.
9. Jones KE, Patel NG, Levy MA, et al. Global trends in emerging infectious diseases. *Nature*. 2008; **451:** 990-93.
10. Miao D, Liu MJ, Wang YX, et al. Epidemiology and ecology of severe fever with thrombocytopenia syndrome in China, 2010‒2018. *Clin Infect Dis*. 2021; **73:** e3851-e3858.
11. Magalhães AR, Codeço CT, Svenning JC, et al. Neglected tropical diseases risk correlates with poverty and early ecosystem destruction. *Infect Dis Poverty*. 2023; **12:** 32.
12. Gallardo B, Zieritz A, Aldridge DC. The importance of the human footprint in shaping the global distribution of terrestrial, freshwater and marine invaders. *PLoS One*. 2015; **10:** e0125801.
13. Skinner EB, Glidden CK, MacDonald AJ, Mordecai EA. Human footprint is associated with shifts in the assemblages of major vector-borne diseases. *Nat Sustain*. 2023; **6:** 652–61.
14. Sulaiman Usman, Ibrahim Isa Adamu. Modeling the transmission dynamics of the monkeypox virus infection with treatment and vaccination interventions. *J App Math Phys*, 2017; **5:** 2335.
15. Olival KJ, Hosseini PR, Zambrana-Torrelio C, Ross N, Bogich TL, Daszak P. Host and viral traits predict zoonotic spillover from mammals. *Nature*. 2017; **546:** 646-50

# Table S7: Variables used for ecological modelling in this study.

| **Classification of variables** | **Variable** | **Description** |
| --- | --- | --- |
| **Ecoclimatic variables** |  |  |
|  | BIO1 | Annual mean temperature (℃) |
|  | BIO2 | Mean diurnal range (Mean of monthly (max temp-min temp)) (℃) |
|  | BIO3 | Isothermality (BIO2/ BIO7) (×100) |
|  | BIO4 | Temperature seasonality (standard deviation×100) |
|  | BIO5 | Max temperature of warmest month (℃) |
|  | BIO6 | Min temperature of coldest month (℃) |
|  | BIO7 | Annual range of temperature (BIO5- BIO6) (℃) |
|  | BIO8 | Mean temperature of wettest quarter (℃) |
|  | BIO9 | Mean temperature of driest quarter (℃) |
|  | BIO10 | Mean temperature of warmest quarter (℃) |
|  | BIO11 | Mean temperature of coldest quarter (℃) |
|  | BIO12 | Annual precipitation (mm) |
|  | BIO13 | Precipitation of wettest month (mm) |
|  | BIO14 | Precipitation of driest month (mm) |
|  | BIO15 | Precipitation seasonality (Coefficient of variation) |
|  | BIO16 | Precipitation of wettest quarter (mm) |
|  | BIO17 | Precipitation of driest quarter (mm) |
|  | BIO18 | Precipitation of warmest quarter (mm) |
|  | BIO19 | Precipitation of coldest quarter (mm) |
| **Environmental variables** |  |  |
|  | Elevation | Average elevation (m) |
|  | Leaf area index | area of leaves (m²) over a unit of land (m²) |
|  | Cropland | Percentage coverage of cropland (%) |
|  | Mixed cropland and nature vegetation | Percentage coverage of mixed cropland and nature vegetation (%) |
|  | Forest | Percentage coverage of forest (%) |
|  | Shrubland | Percentage coverage of shrubland (%) |
|  | Mixed tree, shrub and herbaceous | Percentage coverage of mixed tree, shrub and herbaceous (%) |
|  | Grassland | Percentage coverage of grassland (%) |
|  | Lichens and mosses | Percentage coverage of lichens and mosses (%) |
|  | Sparse vegetation land | Percentage coverage of sparse vegetation land (%) |
|  | Flooded vegetation | Percentage coverage of flooded vegetation (%) |
| **Classification of variables** | **Variable** | **Description** |
|  | Urban built-up land | Percentage coverage of urban construction land (%) |
|  | Bare areas | Percentage coverage of bare areas (%) |
|  | Water body | Percentage coverage of inland water body (%) |
|  | Ice and snow | Percentage coverage of ice and snow (%) |
| **Biological variables** |  |  |
|  | Buffalo | Density of buffalo (heads per km²) |
|  | Cattle | Density of cattle (heads per km²) |
|  | Goat | Density of goat (heads per km²) |
|  | Sheep | Density of sheep (heads per km²) |
|  | Horse | Density of horse (heads per km²) |
|  | Mammalian richness | The number of mammal species per km² |
|  | Rodent richness | The number of rodent species per km² |
| **Socioeconomic variables** |  |  |
|  | Population number | Average of population counts per km² |
|  | Global downscaled GDP | The assessment of GDP per raster |
|  | Human Footprint^#^ | The annual dynamics of the global human footprint per km² |

# Table S8: The number of studies and positive occurrence grids for the ten *Babesia* species with reports of confirmed human infection.

| ***Babesia* species** | **Number of studies** | **Number of positive occurrence grids** | **Number of grids can used in the modeling analysis** |
| --- | --- | --- | --- |
| *Babesia microti** | 349 | 806 | 591 |
| *Babesia* sp. venatorum*** | 132 | 370 | 304 |
| *Babesia bigemina** | 115 | 313 | 164 |
| *Babesia divergens** | 98 | 230 | 150 |
| *Babesia bovis** | 88 | 249 | 107 |
| *Babesia odocoilei** | 25 | 82 | 67 |
| *Babesia duncani* | 8 | 19 | 3 |
| *Babesia sp. FR1* | 1 | 1 | 1 |
| *Babesia sp. KO1* | 1 | 1 | 0 |
| *Babesia sp. XXB/HangZhou* | 1 | 1 | 0 |

* Six major species of *Babesia* were included in the niche model based on the above data.

# Table S9: The tick species carrying the six major *Babeisa* species.

| Species | *Babesia microti* | | *Babesia bigemina* | | | *Babesia* sp. venatorum | | | *Babesia divergens* | | | *Babesia odocoilei* | | | *Babesia bovis* | | |  |
| --- | --- | --- | --- | --- | --- | --- | --- | --- | --- | --- | --- | --- | --- | --- | --- | --- | --- | --- |
|  | N | R (95% CI) | | N | R (95% CI) | | N | R (95% CI) | | N | R (95% CI) | | N | R (95% CI) | | N | R (95% CI) | |
| *A. lepidum* |  |  | |  |  | |  |  | |  |  | |  |  | |  |  | |
| *C. vespertilionis* |  |  | |  |  | | 1 | 0·0083 | |  |  | |  |  | |  |  | |
| *D. marginatus* |  |  | |  |  | |  |  | | 1 | 0·0043 | |  |  | |  |  | |
| *D. nuttalli* |  |  | |  |  | | 1 | 0·0394 | |  |  | |  |  | |  |  | |
| *D. reticulatus** | 6 | 0·0250 (0·0115‒0·0534) | |  |  | | 2 | 0·0026 (0·0006‒0·0104) | | 1 | 0·0265 | |  |  | | 2 | 0·0104 (0·0035‒0·0313) | |
| *D. silvarum* |  |  | |  |  | | 1 | 0·0163 | |  |  | |  |  | |  |  | |
| *Ha. concinna* |  |  | | 1 | 0·0026 | |  |  | | 1 | 0·0017 | |  |  | |  |  | |
| *Ha. longicornis* | 3 | 0·0046 (0·0014‒0·0146) | |  |  | |  |  | | 1 | 0·0068 | |  |  | |  |  | |
| *Ha. punctata* |  |  | | 1 | 0·009 | |  |  | |  |  | |  |  | |  |  | |
| *Hy. anatolicum* |  |  | |  |  | |  |  | |  |  | |  |  | |  |  | |
| *Hy. excavatum* |  |  | |  |  | |  |  | |  |  | |  |  | |  |  | |
| *Hy. impeltatum* |  |  | |  |  | |  |  | |  |  | |  |  | |  |  | |
| *Hy. marginatum* | 1 | 0·0183 | |  |  | |  |  | |  |  | |  |  | |  |  | |
| *Hy. rufipes* |  |  | |  |  | |  |  | |  |  | |  |  | |  |  | |
| *I. canisuga* | 1 | 0·0023 | |  |  | |  |  | |  |  | | 1 | 0·0023 | |  |  | |
| *I. hexagonus* | 1 | 0·0035 | |  |  | |  |  | |  |  | |  |  | |  |  | |
| *I. ovatus* | 3 | 0·0766 (0·0196‒0·2565) | |  |  | |  |  | |  |  | |  |  | |  |  | |
| *I. pacificus* |  |  | |  |  | |  |  | |  |  | |  |  | |  |  | |
| *I. pavlovskyi* | 1 | 0·0093 | |  |  | |  |  | |  |  | |  |  | |  |  | |
| *I. persulcatus** | 13 | 0·0153 (0·0076‒0·0306) | | 1 | 0·0025 | | 10 | 0·0116 (0·0061‒0·0218) | | 4 | 0·0067 (0·0026‒0·0174) | |  |  | |  |  | |
| *I. ricinus** | 62 | 0·0178 (0·0128‒0·0245) | | 1 | 0·0052 | | 70 | 0·0114 (0·0093‒0·0139) | | 30 | 0·0055 (0·0034‒0·0087) | | 1 | 0·0222 | |  |  | |
| *I. scapularis** | 24 | 0·0504 (0·0342‒0·0736) | |  |  | |  |  | |  |  | | 9 | 0·0763 (0·0556‒0·1047) | |  |  | |
| *R. Annulatus* |  |  | | 1 | 0·0054 | |  |  | |  |  | | 1 | 0·0511 | | 1 | 0·0043 | |
| Species | *Babesia microti* | | | *Babesia bigemina* | | | *Babesia* sp. venatorum | | | *Babesia divergens* | | | *Babesia odocoilei* | | | *Babesia bovis* | | |
|  | N | R (95% CI) | | N | R (95% CI) | | N | R (95% CI) | | N | R (95% CI) | | N | R (95% CI) | | N | R (95% CI) | |
| *R. decoloratus* |  |  | | 2 | 0·0136 (0·0003‒0·6125) | |  |  | |  |  | |  |  | | 1 | 0·009 | |
| *R. evertsi* |  |  | | 1 | 0·0004 | |  |  | |  |  | |  |  | |  |  | |
| *R. microplus** | 1 | 0·0278 | | 4 | 0·0402 (0·0069‒0·2341) | |  |  | |  |  | |  |  | |  |  | |
| *R. praetextatus* |  |  | |  |  | |  |  | |  |  | |  |  | |  |  | |
| *R. sanguineus* | 1 | 0·0232 | |  |  | | 1 | 0·0643 | |  |  | |  |  | |  |  | |

*Five tick species were determined as the main vectors of the six *Babesia* species based on the number of studies and proportion of detecting with *Babesia* in ticks, which were included in the niche model. N represents the number of studies detecting *Babesia* in ticks. R represents the correlation coefficient.

# Table S10: The number of studies and occurrence locations for the two tick species.

| **Tick species** | **Number of studies collected** | **Number of studies included** | **Number of locations collected** | **Number of locations used in modelling analysis** |
| --- | --- | --- | --- | --- |
| *Dermacentor reticulatus* | 597 | 53 | 272 | 210 |
| *Rhipicephalus microplus* | 1 524 | 166 | 3 183 | 2 693 |

# Table S11: Vectors with evidence of biting human

| **Vector** | **Species** | **Reference** |
| --- | --- | --- |
| **Tick** | *Amblyomma americanum* | Madison-Antenucci S, Kramer LD, Gebhardt LL, Kauffman E. Emerging Tick-Borne Diseases. Clin Microbiol Rev 2020; 33(2). |
|  | *Amblyomma variegatum* | Petney TN, Horak IG, Rechav Y. The ecology of the African vectors of heartwater, with particular reference to Amblyomma hebraeum and Amblyomma variegatum. Onderstepoort J Vet Res 1987; 54(3): 381-95. |
|  | Amblyomma cajennense | Szabó MP, Pinter A, Labruna MB. Ecology, biology and distribution of spotted-fever tick vectors in Brazil. Front Cell Infect Microbiol 2013; 3: 27. |
|  | *Amblyomma testudinarium* | Chao LL, Lu CW, Lin YF, Shih CM. Molecular and morphological identification of a human biting tick, Amblyomma testudinarium (Acari: Ixodidae), in Taiwan. Exp Appl Acarol 2017; 71(4): 401-14. |
|  | *Carios vespertilionis* | Jaenson TGT, Wilhelmsson P. First Record of a Suspected Human-Pathogenic Borrelia Species in Populations of the Bat Tick Carios vespertilionis in Sweden. Microorganisms. 2021 May 20;9(5):1100. |
|  | *Dermacentor variabilis* | Madison-Antenucci S, Kramer LD, Gebhardt LL, Kauffman E. Emerging Tick-Borne Diseases. Clin Microbiol Rev 2020; 33(2). |
|  | *Dermacentor marginatus* | Garcia-Vozmediano A, Giglio G, Ramassa E, Nobili F, Rossi L, Tomassone L. Dermacentor marginatus and Dermacentor reticulatus, and Their Infection by SFG Rickettsiae and Francisella-Like Endosymbionts, in Mountain and Periurban Habitats of Northwestern Italy. Vet Sci 2020; 7(4). |
|  | *Dermacentor nuttalli* | Khasnatinov MA, Liapunov AV, Manzarova EL, Kulakova NV, Petrova IV, Danchinova GA. The diversity and prevalence of hard ticks attacking human hosts in Eastern Siberia (Russian Federation) with first description of invasion of non-endemic tick species. Parasitol Res 2016; 115(2): 501-10. |
|  | *Dermacentor reticulatus* | Khasnatinov MA, Liapunov AV, Manzarova EL, Kulakova NV, Petrova IV, Danchinova GA. The diversity and prevalence of hard ticks attacking human hosts in Eastern Siberia (Russian Federation) with first description of invasion of non-endemic tick species. Parasitol Res 2016; 115(2): 501-10. |
|  | *Dermacentor silvarum* | Guo WB, Shi WQ, Wang Q, et al. Distribution of Dermacentor silvarum and Associated Pathogens: Meta-Analysis of Global Published Data and a Field Survey in China. Int J Environ Res Public Health 2021; 18(9). |
|  | *Haemaphysalis longicornis* | Madison-Antenucci S, Kramer LD, Gebhardt LL, Kauffman E. Emerging Tick-Borne Diseases. Clin Microbiol Rev 2020; 33(2). |
|  | *Haemaphysalis punctata* | Raad M, Azar D, Perotti MA. First Report of the Ticks Haemaphysalis punctata Canestrini et Fanzago, 1878, Haemaphysalis parva (Neumann, 1897) and Dermacentor marginatus (Sulzer, 1776) (Acari, Amblyommidae) from Humans in Lebanon. Acta Parasitol 2020; 65(2): 541-5. |
|  | *Haemaphysalis concinna* | Khasnatinov MA, Liapunov AV, Manzarova EL, Kulakova NV, Petrova IV, Danchinova GA. The diversity and prevalence of hard ticks attacking human hosts in Eastern Siberia (Russian Federation) with first description of invasion of non-endemic tick species. Parasitol Res 2016; 115(2): 501-10. |
|  | *Haemaphysalis erinacei* | Keskin A, Keskin A, Bursali A, Tekin S. Ticks (Acari: Ixodida) parasitizing humans in Corum and Yozgat provinces, Turkey. Exp Appl Acarol 2015; 67(4): 607-16. |
|  | *Haemaphysalis parva* | Keskin A, Keskin A, Bursali A, Tekin S. Ticks (Acari: Ixodida) parasitizing humans in Corum and Yozgat provinces, Turkey. Exp Appl Acarol 2015; 67(4): 607-16. |
|  | *Haemaphysalis sulcata* | Keskin A, Keskin A, Bursali A, Tekin S. Ticks (Acari: Ixodida) parasitizing humans in Corum and Yozgat provinces, Turkey. Exp Appl Acarol 2015; 67(4): 607-16. |
|  | *Haemaphysalis flava* | Ozawa A, Yamaguchi N, Hayakawa K, Matsuo I, Niizuma K, Ohkido M. [A case of tick bite (Haemaphysalis flava)--consideration of tularemia infection through tick bite]. Nihon Hifuka Gakkai Zasshi 1982; 92(13): 1415-21. |
|  | *Haemaphysalis japonica* | Sasaki K, Honma M, Nakao M, et al. Survey to detect tick-borne encephalitis virus from human-feeding ticks in Hokkaido, Japan. J Dermatol 2021; 48(7): 1094-7. |
|  | *Haemaphysalis leachi* | Dick G, Lewis E. A rickettsial disease in East Africa transmitted by ticks (Rhipicephalus simus and Haemaphysalis leachi). Transactions of the Royal Society of Tropical Medicine and Hygiene 1947; 41(3): 295-326. |
|  | *Haemaphysalis leporispalustris* | Hahn MB, Disler G, Durden LA, et al. Establishing a baseline for tick surveillance in Alaska: Tick collection records from 1909-2019. Ticks Tick Borne Dis 2020; 11(5): 101495. |
| **Vector** | **Species** | **Reference** |
|  | *Haemaphysalis bancrofti* | Gofton AW, Doggett S, Ratchford A, et al. Bacterial Profiling Reveals Novel "Ca. Neoehrlichia", Ehrlichia, and Anaplasma Species in Australian Human-Biting Ticks. PLoS One. 2015;10(12):e0145449. |
|  | *Hyalomma excavatum* | Keskin A, Keskin A, Bursali A, Tekin S. Ticks (Acari: Ixodida) parasitizing humans in Corum and Yozgat provinces, Turkey. Exp Appl Acarol 2015; 67(4): 607-16. |
|  | *Haemaphysalis qinghaiensis* | Guglielmone, A.A., Robbins, R.G.Tick Species Found Feeding on Humans.2018. |
|  | *Hyalomma marginatum* | Keskin A, Keskin A, Bursali A, Tekin S. Ticks (Acari: Ixodida) parasitizing humans in Corum and Yozgat provinces, Turkey. Exp Appl Acarol 2015; 67(4): 607-16. |
|  | *Hyalomma anatolicum* | Hosseini A, Dalimi A, Abdigoudarzi M. Morphometric Study on Male Specimens of Hyalomma anatolicum (Acari: Ixodidae) in West of Iran. Iran J Arthropod Borne Dis 2011; 5(2): 23-31. |
|  | *Hyalomma asiaticum* | Liu ZQ, Xia J, Wang GL, Kuermanali N. Cloning and expression of the 4D8 gene from Hyalomma asiaticum tick. Genet Mol Res 2016; 15(2). |
|  | *Hyalomma dromedarii* | Mosabah AA, Morsy TA. Tick paralysis: first zoonosis record in Egypt. J Egypt Soc Parasitol 2012; 42(1): 71-8. |
|  | *Hyalomma rufipes* | Medialdea-Carrera R, Melillo T, Micaleff C, Borg ML. Detection of Hyalomma rufipes in a recently arrived asylum seeker to the EU. Ticks Tick Borne Dis 2021; 12(1): 101571. |
|  | *Hyalomma scupense* | Kar S, Dervis E, Akın A, Ergonul O, Gargili A. Preferences of different tick species for human hosts in Turkey. Exp Appl Acarol 2013; 61(3): 349-55. |
|  | *Hyalomma impeltatum* | Bursali A, Keskin A, Tekin S. Ticks (Acari: Ixodida) infesting humans in the provinces of Kelkit Valley, a Crimean-Congo Hemorrhagic Fever endemic region in Turkey. Exp Appl Acarol 2013; 59(4): 507-15. |
|  | *Ixodes persulcatus* | Madison-Antenucci S, Kramer LD, Gebhardt LL, Kauffman E. Emerging Tick-Borne Diseases. Clin Microbiol Rev 2020; 33(2). |
|  | *Ixodes ricinus* | Madison-Antenucci S, Kramer LD, Gebhardt LL, Kauffman E. Emerging Tick-Borne Diseases. Clin Microbiol Rev 2020; 33(2). |
|  | *Ixodes scapularis* | Madison-Antenucci S, Kramer LD, Gebhardt LL, Kauffman E. Emerging Tick-Borne Diseases. Clin Microbiol Rev 2020; 33(2). |
|  | *Ixodes ovatus* | Sasaki K, Honma M, Nakao M, et al. Survey to detect tick-borne encephalitis virus from human-feeding ticks in Hokkaido, Japan. J Dermatol 2021; 48(7): 1094-7. |
|  | *Ixodes pacificus* | David T Gauthier, Christopher D Paddock, et al. Characterization of a novel transitional group Rickettsia species (Rickettsia tillamookensis sp. nov.) from the western black-legged tick, Ixodes pacificus. Int J Syst Evol Microbiol 2021; 71(7): 004880. |
|  | *Ixodes cookei* | Eisen L. Tick species infesting humans in the United States. Ticks Tick Borne Dis. 2022;13(6):102025. |
|  | *Ixodes angustus* | Eisen L. Tick species infesting humans in the United States. Ticks Tick Borne Dis. 2022;13(6):102025. |
|  | *Ixodes simplex* | Péter Á, Barti L, Corduneanu A, Hornok S, Mihalca AD, Sándor AD. First record of Ixodes simplex found on a human host, with a review of cases of human infestation by bat tick species occurring in Europe. Ticks Tick Borne Dis. 2021;12(4):101722. |
|  | *Ixodes hexagonus* | Faulde MK, Rutenfranz M, Hepke J, Rogge M, Görner A, Keth A. Human tick infestation pattern, tick-bite rate, and associated Borrelia burgdorferi s.l. infection risk during occupational tick exposure at the Seedorf military training area, northwestern Germany. Ticks Tick Borne Dis 2014; 5(5): 594-9. |
|  | *Ixodes trianguliceps* | Hubbard MJ, Baker AS, Cann KJ. Distribution of Borrelia burgdorferi s.l. spirochaete DNA in British ticks (Argasidae and Ixodidae) since the 19th century, assessed by PCRhipicephalus Med Vet Entomol 1998; 12(1): 89-97. |
|  | *Rhipicephalus sanguineus* | Madison-Antenucci S, Kramer LD, Gebhardt LL, Kauffman E. Emerging Tick-Borne Diseases. Clin Microbiol Rev 2020; 33(2). |
| **Vector** | **Species** | **Reference** |
|  | *Rhipicephalus microplus* | Szabó MPJ, Martins TF, Barbieri ARM, et al. Ticks biting humans in the Brazilian savannah: Attachment sites and exposure risk in relation to species, life stage and season. Ticks Tick Borne Dis 2020; 11(2): 101328. |
|  | *Rhipicephalus annulatus* | Keskin A, Keskin A, Bursali A, Tekin S. Ticks (Acari: Ixodida) parasitizing humans in Corum and Yozgat provinces, Turkey. Exp Appl Acarol 2015; 67(4): 607-16. |
|  | *Rhipicephalus bursa* | Keskin A, Keskin A, Bursali A, Tekin S. Ticks (Acari: Ixodida) parasitizing humans in Corum and Yozgat provinces, Turkey. Exp Appl Acarol 2015; 67(4): 607-16. |
|  | *Rhipicephalus turanicus* | Keskin A, Keskin A, Bursali A, Tekin S. Ticks (Acari: Ixodida) parasitizing humans in Corum and Yozgat provinces, Turkey. Exp Appl Acarol 2015; 67(4): 607-16. |
|  | *Rhipicephalus simus* | Dick G, Lewis E. A rickettsial disease in East Africa transmitted by ticks (Rhipicephalus simus and Haemaphysalis leachi). Transactions of the Royal Society of Tropical Medicine and Hygiene 1947; 41(3): 295-326. |
|  | *Rhipicephalus appendiculatus* | Horak IG, Fourie LJ, Heyne H, Walker JB, Needham GRhipicephalus Ixodid ticks feeding on humans in South Africa: with notes on preferred hosts, geographic distribution, seasonal occurrence and transmission of pathogens. Exp Appl Acarol 2002; 27(1-2): 113-36. |
|  | *Rhipicephalus evertsi* | Horak IG, Fourie LJ, Heyne H, Walker JB, Needham GRhipicephalus Ixodid ticks feeding on humans in South Africa: with notes on preferred hosts, geographic distribution, seasonal occurrence and transmission of pathogens. Exp Appl Acarol 2002; 27(1-2): 113-36. |
|  | *Rhipicephalus haemaphysaloides* | Soundararajan C, Nagarajan K, Arul Prakash M. Tick infestation in human beings in the Nilgiris and Kancheepuram district of Tamil Nadu, India. J Parasit Dis 2018; 42(1): 50-4. |
|  | *Dermacentor nitens* | Szabó MPJ, Martins TF, Barbieri ARM, et al. Ticks biting humans in the Brazilian savannah: Attachment sites and exposure risk in relation to species, life stage and season. Ticks Tick Borne Dis. 2020;11(2):101328. |
|  | *Hyalomma aegyptium* | Keskin A, Keskin A, Bursali A, Tekin S. Ticks (Acari: Ixodida) parasitizing humans in Corum and Yozgat provinces, Turkey. Exp Appl Acarol. 2015;67(4):607-616. |
|  | *Hyalomma lusitanicum* | Santos-Silva MM, Beati L, Santos AS, et al. The hard-tick fauna of mainland Portugal (Acari: Ixodidae): an update on geographical distribution and known associations with hosts and pathogens. Exp Appl Acarol. 2011;55(1):85-121. |
|  | *Ixodes tasmani* | Guglielmone, A.A., & Robbins, R.G. (2018). Tick Species Found Feeding on Humans. |
|  | *Ixodes ventalloi* | Sanogo YO, Parola P, Shpynov S, et al. Genetic diversity of bacterial agents detected in ticks removed from asymptomatic patients in northeastern Italy. Ann N Y Acad Sci. 2003;990:182-190. |
| Flea | *Ctenocephalides canis* | Beck W, Clark HH. [Differential diagnosis of medically relevant flea species and their significance in dermatology]. Hautarzt 1997; 48(10): 714-9. |
|  | *Ctenocephalides felis* | Mumcuoglu Y, Rufli T. [Siphonaptera/fleas (author's transl)]. Schweiz Rundsch Med Prax 1979; 68(37): 1172-82. |

# Table S12: The co-infection of *Babesia* species and their infected vectors.

| ***Babesia* 1** | ***Babesia* 2** | **Detected vectors** |
| --- | --- | --- |
| *Babesia bigemina* | *Babesia divergens* | *Haemaphysalis punctata* |
| *Babesia bigemina* | *Babesia annae* | *Ixodes ricinus* |
| *Babesia divergens* | *Babesia caballi* | *Dermacentor reticulatus* |
| *Babesia microti* | *Babesia canis* | *Dermacentor reticulatus* |
| *Babesia microti* | *Babesia odocoilei* | *Ixodes scapularis* |
| *Babesia* sp. venatorum | *Babesia capreoli* | *Ixodes ricinus* |

# Table S13: The number of human cases of *Babesia* infection*.*

| **Pathogenic *Babesia*** | **Confirmed cases*** | **Cases with reported clinical manifestations** |
| --- | --- | --- |
| Overall | 26 848 (100) | 706 (100) |
| *Babesia microti* | 25 576 (95·3) | 622 (88·1) |
| *Babesia* sp. venatorum | 58 (0·2) | 40 (5·7) |
| *Babesia bigemina* | 41 (0·2) | 2 (~0·3) |
| *Babesia bovis* | 4 (~0) | 4 (~0·6) |
| *Babesia divergens* | 31 (0·1) | 29 (4·1) |
| *Babesia odocoilei* | 2 (~0) | 2 (~0·3) |
| *Babesia duncani* | 1133 (4·2) | 4 (~0·6) |
| *Babesia* sp. XXB/HangZhou | 1 (~0) | 1 (~0·1) |
| *Babesia* sp. KO1 | 1 (~0) | 1 (~0·1) |
| *Babesia* sp. FR1 | 1 (~0) | 1 (~0·1) |

*Data are presented as numbers of positive infections and proportions (%).

**Table S14: Clinical characteristics of human infections with *Babesia* species.**

| **Symptoms** | ***Babesia microti***  **(n=622)** | ***Babesia* sp. venatorum**  **(n=40)** | ***Babesia divergens***  **(n=29)** |
| --- | --- | --- | --- |
| **Influenza-like symptom** |  |  |  |
| Fever | 494 (79.4) | 26 (65.0) | 17 (58.6) |
| Fatigue | 356‒361 (57.2‒58.0) | 16 (40.0) | 5 (17.2) |
| Chills | 312 (50.2) | 4 (10.0) | 4 (13.8) |
| Sweats | 252‒278 (40.5‒44.7) |  |  |
| Malaise | 187‒200 (30.1‒32.2) | 1‒3 (2.5‒7.5) | 2 (6.9) |
| Headaches | 149‒153 (24.0‒24.6) | 15 (37.5) | 3 (10.3) |
| Weakness | 149‒168 (24.0‒27.0) | 3‒5 (7.5‒12.5) |  |
| Cough | 115‒124 (18.5‒19.9) |  | 2 (6.9) |
| Dizziness | 63‒69 (10.1‒11.1) | 9 (22.5) |  |
| **Motor system symptoms** |  |  |  |
| Myalgia | 179‒186 (28.8‒29.9) | 7 (17.5) | 10 (34.5) |
| Arthralgia | 177‒183 (28.5‒29.4) | 7 (17.5) | 2 (6.9) |
| **Gastrointestinal symptoms** |  |  |  |
| Anorexia | 208‒214 (33.4‒34.4) |  | 3 (10.3) |
| Nausea | 177‒179 (28.5‒28.8) | 6 (15.0) |  |
| Vomit | 91‒96 (14.6‒15.4) |  |  |
| Abdominal pain | 58‒71 (9.3‒11.4) |  | 4 (13.8) |
| Diarrhea | 42‒51 (6.8‒8.2) |  | 1 (3.4) |
| **Neurological symptoms** |  |  |  |
| Malaise | 187‒200 (30.1‒32.2) | 1‒3 (2.5‒7.5) | 2 (6.9) |
| Hypersomnia | 8‒38 (1.3‒6.1) | 8 (20.0) |  |
| neck stiffness | 35‒65 (5.6‒10.5) |  |  |
| **Other symptoms** |  |  |  |
| Rash | 48‒58 (7.7‒9.3) | 6 (15.0) |  |
| Dark urine | 23‒45 (3.7‒7.2) | 3‒5 (7.5‒12.5) | 6 (20.7) |
| Dyspnea | 77‒90 (12.4‒14.5) | 2‒4 (5.0‒10.0) | 1 (3.4) |
| Jaundice | 3‒36 (0.5‒5.8) | 1‒3 (2.5‒7.5) | 4 (13.8) |
| Splenomegaly | 14‒36 (2.3‒5.8) |  |  |
| Sore throat | 23‒54 (3.7‒8.7) |  |  |

Data are presented as numbers of positive cases and proportions (%). Symptoms of less than 10 cases in all *Babesia* species were not showed.

# Table S15: The RCs of significant contributors to the occurrence of two main species of ticks based on BRT models.

| **Variable** | ***D. reticulatus*** | ***R. microplus*** |
| --- | --- | --- |
| Annual mean temperature | 3.9 (3.5‒4.3) |  |
| Temperature seasonality | 9.1 (8.7‒9.5) | 15.7 (15.5‒15.9) |
| Annual range of temperature | 3.5 (3.2‒3.8) |  |
| Mean temperature of wettest quarter | 12.3 (11.6‒13.1) |  |
| Min temperature of coldest month | 3.4 (3.1‒3.7) |  |
| Precipitation of wettest month |  | 11.8 (11.5‒12.1) |
| Precipitation of warmest quarter | 4.8 (4.6‒5.1) | 5.4 (5.3‒5.5) |
| Urban built-up land | 6.7 (6.4‒6.9) | 4.9 (4.9‒5.0) |
| Grass | 4.0 (3.8‒4.1) |  |
| Elevation | 4.4 (4.2‒4.6) |  |
| Cattle |  | 9.6 (9.4‒9.8) |
| Horse |  | 8.2 (8.1‒8.3) |
| Mammalian richness | 13.4 (12.7‒14.1) | 3.5 (3.4‒3.5) |

The mean (95% CI) RCs of variables in model were displayed. We only demonstrated effects with mean RC >3%. RC, relative contribution.

**Table S16: The VIF values for each variable in the models of *Babesia*.**

| **Variables** | ***Babesia microti*** | ***Babesia* sp. venatorum** | ***Babesia divergens*** | ***Babesia bigemina*** | ***Babesia bovis*** | ***Babesia odocoilei*** |
| --- | --- | --- | --- | --- | --- | --- |
| Mean diurnal range | 4.3 | 6.1 | 6.4 | 3.6 | 2.8 | 4.4 |
| Isothermality | - | 4.9 | - | - | 7.2 | - |
| Temperature seasonality | 4 | - | 4.9 | 4.4 | - | - |
| Max temperature of warmest month | 7.8 | 6.4 | 8.2 | 5.9 | - | - |
| Mean temperature of wettest quarter | - | - | - | - | 4.8 | - |
| Precipitation of wettest month | 5.3 | 4.6 | 5.2 | 4.0 | - | 7.5 |
| Mean temperature of driest quarter | - | - | - | - | 5.1 | 5.2 |
| Precipitation of wettest month | - | 7 | - | - | 8 | - |
| Precipitation of driest month | 3.6 | 6 | 3.4 | 3.4 | 5.9 | 6.5 |
| Precipitation seasonality | 3 | 5.2 | 3.6 | 2.5 | 3.2 | 5.3 |
| Precipitation of warmest quarter | 2.7 | 5.8 | 4 | 2.8 | 7.4 | 4.6 |
| Precipitation of coldest quarter | 3.2 | 6.2 | 2 | 2.8 | 5.4 | 3.7 |
| Elevation | 3.8 | 4.1 | 3.4 | 3.4 | 2.6 | 4.2 |
| Leaf area index | 4.3 | 4.3 | 5.7 | 4.4 | 5.3 | 4.7 |
| Cropland | 2.7 | 3.5 | 3 | 3.5 | 3.7 | 2.6 |
| Mixed cropland and nature vegetation | 1.4 | 1.4 | 1.4 | 1.5 | 1.6 | 2 |
| Shrubland | 1.3 | 1.3 | 1.4 | 1.2 | 1.4 | 1.4 |
| Shrub | 1.8 | 1.3 | 1.8 | 2.5 | 2.2 | 2.1 |
| Grass | 1.8 | 2.3 | 1.9 | 1.8 | 2 | 1.7 |
| Lichen_mosses | 1.4 | 1.1 | 1.2 | 1.1 | 1.2 | 1.8 |
| Sparse_vegetation | 1.6 | 1.7 | 1.7 | 1.7 | 1.9 | 2 |
| Flooded vegetation | 1.2 | 1.3 | 1.2 | 1.3 | 1.4 | 1.2 |
| Urban built-up land | 3.9 | 6 | 5.7 | 4 | 7.4 | 7.6 |
| Bare areas | 3.3 | 3.8 | 4.7 | 3.7 | 4.6 | 1.5 |
| **Variables** | ***Babesia microti*** | ***Babesia* sp. venatorum** | ***Babesia divergens*** | ***Babesia bigemina*** | ***Babesia bovis*** | ***Babesia odocoilei*** |
| Water body | 1.9 | 1.7 | 1.7 | 1.6 | 1.9 | 2.2 |
| Ice and snow | 1.4 | 1.2 | 1.2 | 1.1 | 1.4 | 4 |
| Buffalo | 1.3 | 1.4 | 1.6 | 1.6 | 1.6 | 1.4 |
| Cattle | 1.7 | 1.9 | 2.2 | 2.6 | 2.4 | 2.3 |
| Goat | 1.7 | 1.7 | 1.5 | 2.2 | 1.9 | 1.5 |
| Sheep | 1.4 | 1.3 | 1.7 | 1.6 | 1.5 | 1.4 |
| Horse | 1.3 | 1.3 | 1.4 | 1.5 | 1.5 | 1.9 |
| Mammalian richness | 5.9 | 8.4 | 7.8 | 6.1 | 8.6 | 9 |
| Rodent richness | 3.7 | 6.1 | 5.3 | 3.8 | 4.3 | 4.9 |
| Habitat suitability index of ticks | 3 | 4.4 | 3.3 | 2.3 | 2.8 | 4.5 |
| Population number | 2.1 | 4.1 | 3.4 | 2 | 4.2 | 4.4 |
| Global downscaled GDP | 3.4 | 4.9 | 3.8 | 2.7 | 2.8 | 4.3 |
| Human Footprint | 4.5 | 6.1 | 4.5 | 3.2 | 3.2 | 4.5 |

Data are presented as numbers of VIF<10.

**Table S17: Comparison of AUC values for the models of *Babesia* species using two area thresholds.**

| ***Babesia* species** | **Threshold** | |
| --- | --- | --- |
|  | **100 km²** | **400 km²** |
| *Babesia microti* | 0.938 (0.914‒0.959) | 0.936 (0.913‒0.955) |
| *Babesia* sp. venatorum | 0.969 (0.953‒0.987) | 0.975 (0.963‒0.979) |
| *Babesia bigemina* | 0.914 (0.859‒0.953) | 0.921(0.865‒0.956) |
| *Babesia bovis* | 0.909 (0.841‒0.954) | 0.927 (0.873‒0.967) |
| *Babesia divergens* | 0.983 (0.966‒0.996) | 0.984 (0.966‒0.995) |
| *Babesia odocoilei* | 0.961 (0.920‒0.997) | 0.959 (0.911‒0.996) |

**Table S18: Comparison of AUC values for the models of *Babesia* species using two sampling methods.**

| ***Babesia* species** | **Sampling method** | |
| --- | --- | --- |
|  | **Background grids** | **Random grids** |
| *Babesia microti* | 0.867 (0.832‒0.896) | 0.936 (0.913‒0.955) |
| *Babesia* sp. venatorum | 0.907 (0.873‒0.938) | 0.975 (0.963‒0.979) |
| *Babesia bigemina* | 0.905 (0.844‒0.954) | 0.921(0.865‒0.956) |
| *Babesia bovis* | 0.844 (0.752‒0.915) | 0.927 (0.873‒0.967) |
| *Babesia divergens* | 0.887 (0.816‒0.945) | 0.984 (0.966‒0.995) |
| *Babesia odocoilei* | 0.867 (0.776‒0.959) | 0.959 (0.911‒0.996) |

**Table S19:** **The RCs of significant contributors to the spatial distribution of the six major *Babesia* species based on RF models.**

| **Variables** | ***Babesia microti*** | ***Babesia* sp. venatorum** | ***Babesia divergens*** | ***Babesia bigemina*** | ***Babesia bovis*** | ***Babesia odocoilei*** |
| --- | --- | --- | --- | --- | --- | --- |
| Mean diurnal range | 6.2 (6.1‒6.3) | 5.2 (5.1‒5.4) |  |  |  |  |
| Isothermality |  | 5.3 (5.2‒5.4) |  |  | 5.9 (5.7‒6.0) |  |
| Temperature seasonality | 6.3 (6.2‒6.4) |  | 6.7 (6.6‒6.8) | 5.4 (5.3‒5.5) |  |  |
| Max temperature of warmest month | 5.2 (5.1‒5.3) | 6.6 (6.5‒6.7) | 4.7 (4.6‒4.8) | 3.7 (3.6‒3.7) |  |  |
| Mean temperature of wettest quarter | 4.4 (4.4‒4.5) | 6.2 (6.1‒6.3) | 5.9 (5.8‒6.0) | 4.0 (3.9‒4.1) | 6.0 (5.8‒6.3) | 10.2 (9.9‒10.4) |
| Mean temperature of driest quarter | 4.9 (4.9‒5.0) |  |  |  | 5.6 (5.4‒5.8) | 9.7 (9.5‒10.0) |
| Precipitation of wettest month |  | 5.7 (5.7‒5.8) |  |  |  |  |
| Precipitation of driest month |  | 7.1 (7.0‒7.1) | 8.5 (8.4‒8.7) | 3.9 (3.8‒4.0) |  | 4.6 (4.3‒4.8) |
| Precipitation seasonality | 5.4 (5.4‒5.5) |  | 4.5 (4.3‒4.6) | 4.1 (4.0‒4.2) |  | 6.4 (6.1‒6.7) |
| Precipitation of warmest quarter | 4.5 (4.4‒4.5) | 5.8 (5.7‒5.9) | 6.3 (6.2‒6.3) | 4.0 (4.0‒4.1) |  | 5.1 (4.9‒5.3) |
| Precipitation of coldest quarter | 4.7 (4.6‒4.8) | 5.4 (5.3‒5.5) | 5.7 (5.5‒5.8) | 5.4 (5.3‒5.5) | 4.8 (4.6‒4.9) |  |
| Elevation | 5.0 (5.0‒5.1) | 4.8 (4.7‒4.9) |  |  |  |  |
| Leaf area index | 4.7 (4.6‒4.7) |  | 4.3 (4.2‒4.4) | 3.6 (3.5‒3.6) | 5.6 (5.4‒5.8) |  |
| Cropland |  |  |  | 5.5 (5.4‒5.6) | 6.1 (5.9‒6.2) |  |
| Grass |  | 5.1 (5.0‒5.3) |  |  |  |  |
| Urban built-up land | 6.6 (6.5‒6.7) | 5.2 (5.1‒5.3) | 5.4 (5.3‒5.5) | 4.5 (4.3‒4.6) | 8.1 (7.9‒8.3) | 4.8 (4.4‒5.2) |
| Buffalo |  |  | 4.6 (4.5‒4.8) | 3.7 (3.6‒3.9) |  | 7.2 (6.8‒7.6) |
| Cattle |  |  |  | 5.1 (5.0‒5.2) | 4.4 (4.2‒4.6) |  |
| Goat | 4.6 (4.5‒4.6) |  | 4.5 (4.4‒4.6) | 4.1 (4.0‒4.3) | 3.7 (3.5‒3.9) |  |
| Sheep | 4.5 (4.5‒4.6) |  |  | 3.9 (3.8‒4.0) |  |  |
| Horse |  |  |  | 4.4 (4.3‒4.5) |  |  |
| Mammalian richness | 4.4 (4.3‒4.4) | 4.9 (4.8‒5.0) | 5.0 (4.9‒5.1) | 5.0 (4.9‒5.1) | 5.0 (4.8‒5.2) |  |
| Rodentia |  |  |  | 4.5 (4.4‒4.6) | 5.5 (5.4‒5.7) | 5.3 (5.1‒5.5) |
| Habitat suitability index of ticks | 11.6 (11.2‒12.1) | 15.5 (14.4‒16.5) | 14.2 (13.6‒14.8) | 8.1 (7.9‒8.4) | 14.1 (13.6‒14.6) | 19.5 (18.7‒20.3) |
| **Variables** | ***Babesia microti*** | ***Babesia* sp. venatorum** | ***Babesia divergens*** | ***Babesia bigemina*** | ***Babesia bovis*** | ***Babesia odocoilei*** |
| Population number | 5.1 (5.0‒5.2) | 6.0 (5.8‒6.2) | 5.5 (5.4‒5.6) | 7.7 (7.4‒7.9) | 12.0 (11.7‒12.4) | 6.4 (6.0‒6.8) |
| Global downscaled GDP | 7.4 (7.3‒7.5) | 6.6 (6.5‒6.6) | 8.9 (8.7‒9.1) | 3.5 (3.4‒3.6) | 5.4 (5.2‒5.6) | 14.9 (14.4‒15.3) |
| Human Footprint | 4.4 (4.3‒4.5) | 4.6 (4.4‒4.7) | 5.5 (5.4‒5.5) | 6.0 (5.9‒6.1) | 7.8 (7.6‒8.1) | 5.9 (5.6‒6.3) |

Figure S1: The distributions of other *Babesia* species. (A) Distribution of 23 *Babesia* species without recording human infection. (B) Number of infection occurrence locations for each *Babesia* species, stratified by continent.

**
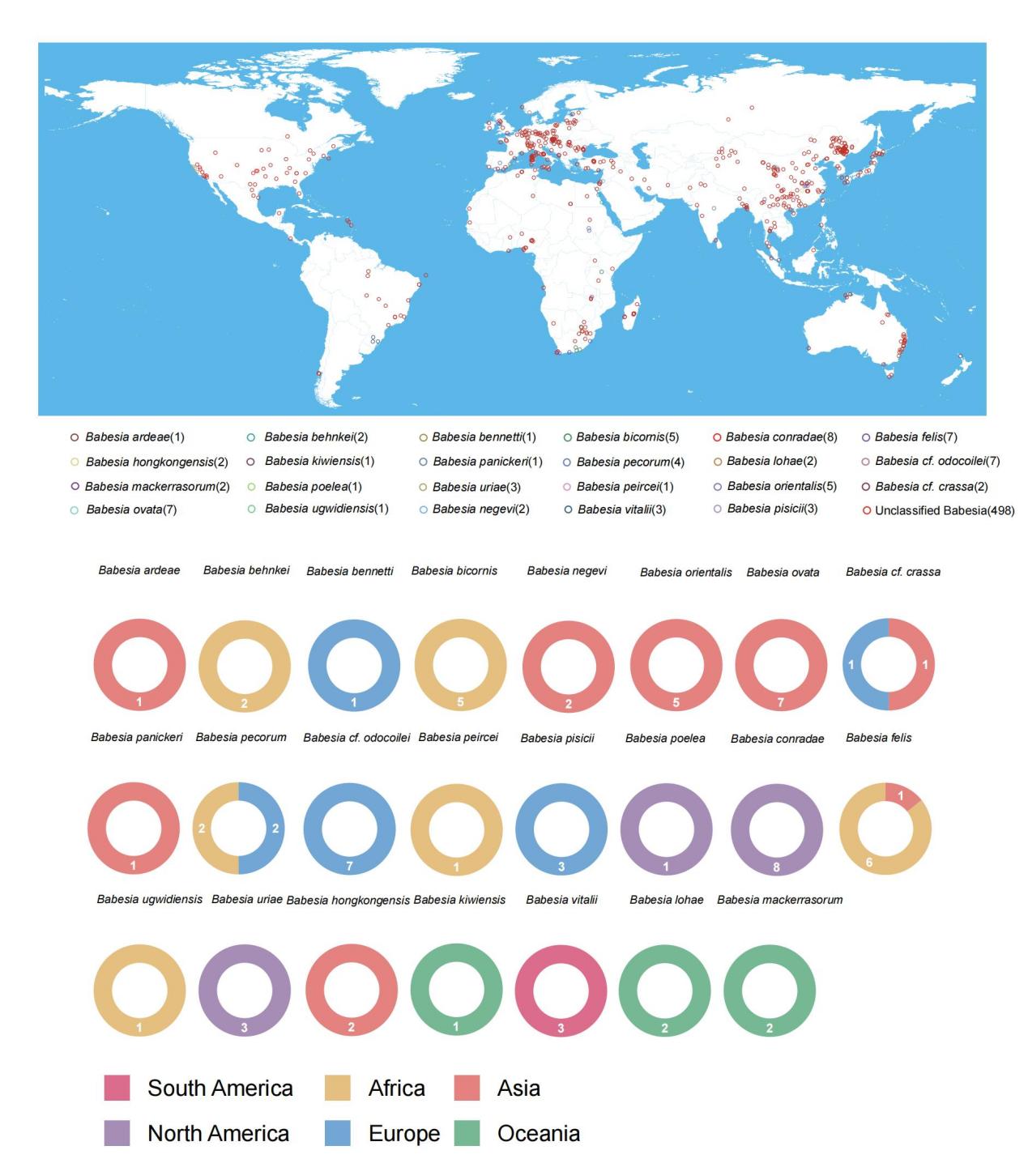
**

Figure S2: Effects of major predictors (RCs >3%) for presence of *Dermacentor reticulatus* based on BRT models. The mean curves (red) and 95% percentiles (blue, biological variables; purple, ecoclimatic variables; green, environmental variables) show the predicted habitat suitability index. The histograms show the frequency distributions of the predictors.


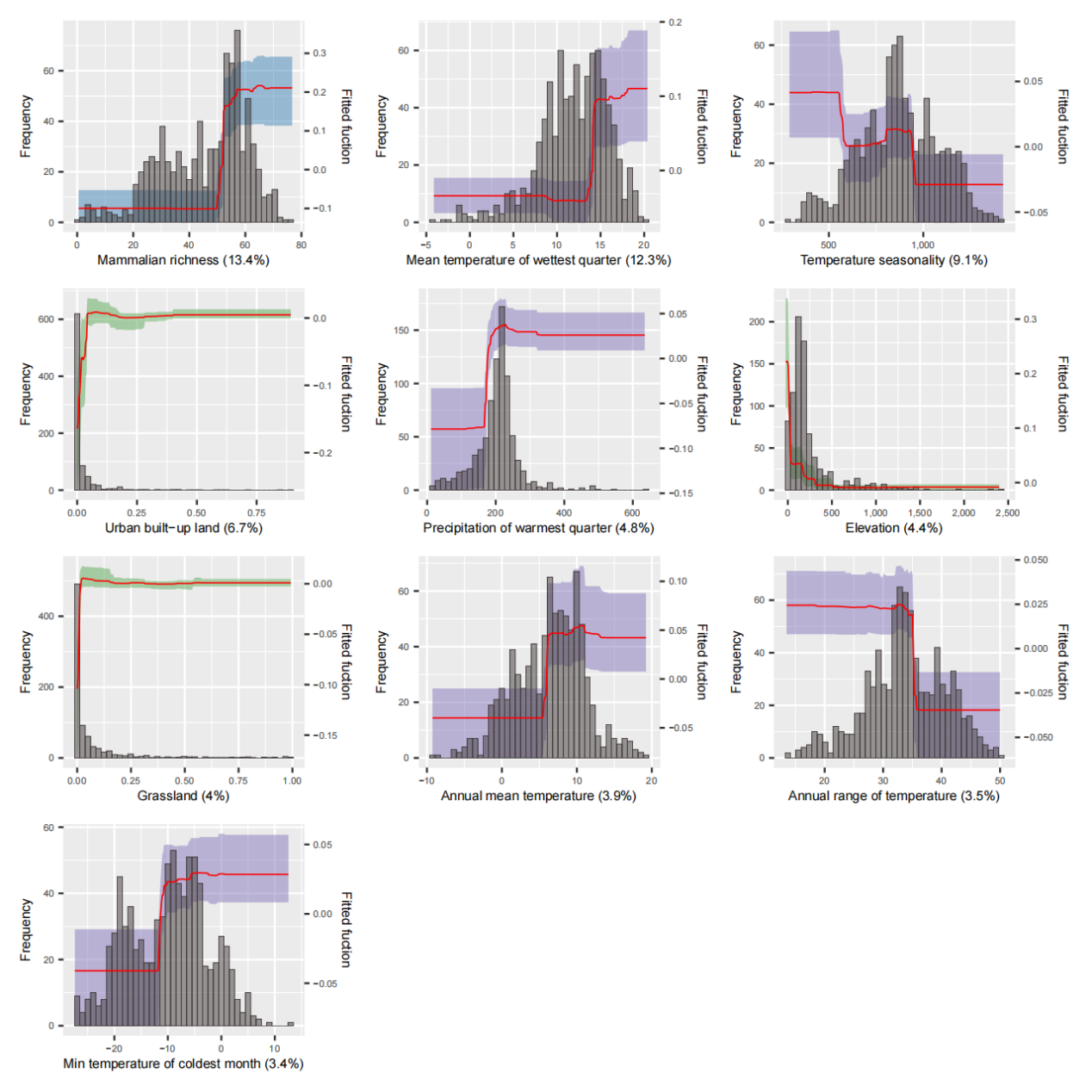


Figure S3: Effects of major predictors (RCs >3%) for presence of *Rhipicephalus microplus* based on BRT models. The mean curves (red) and 95% percentiles (blue, biological variables; purple, ecoclimatic variables; green, environmental variables) show the predicted habitat suitability index. The histograms show the frequency distributions of the predictors.


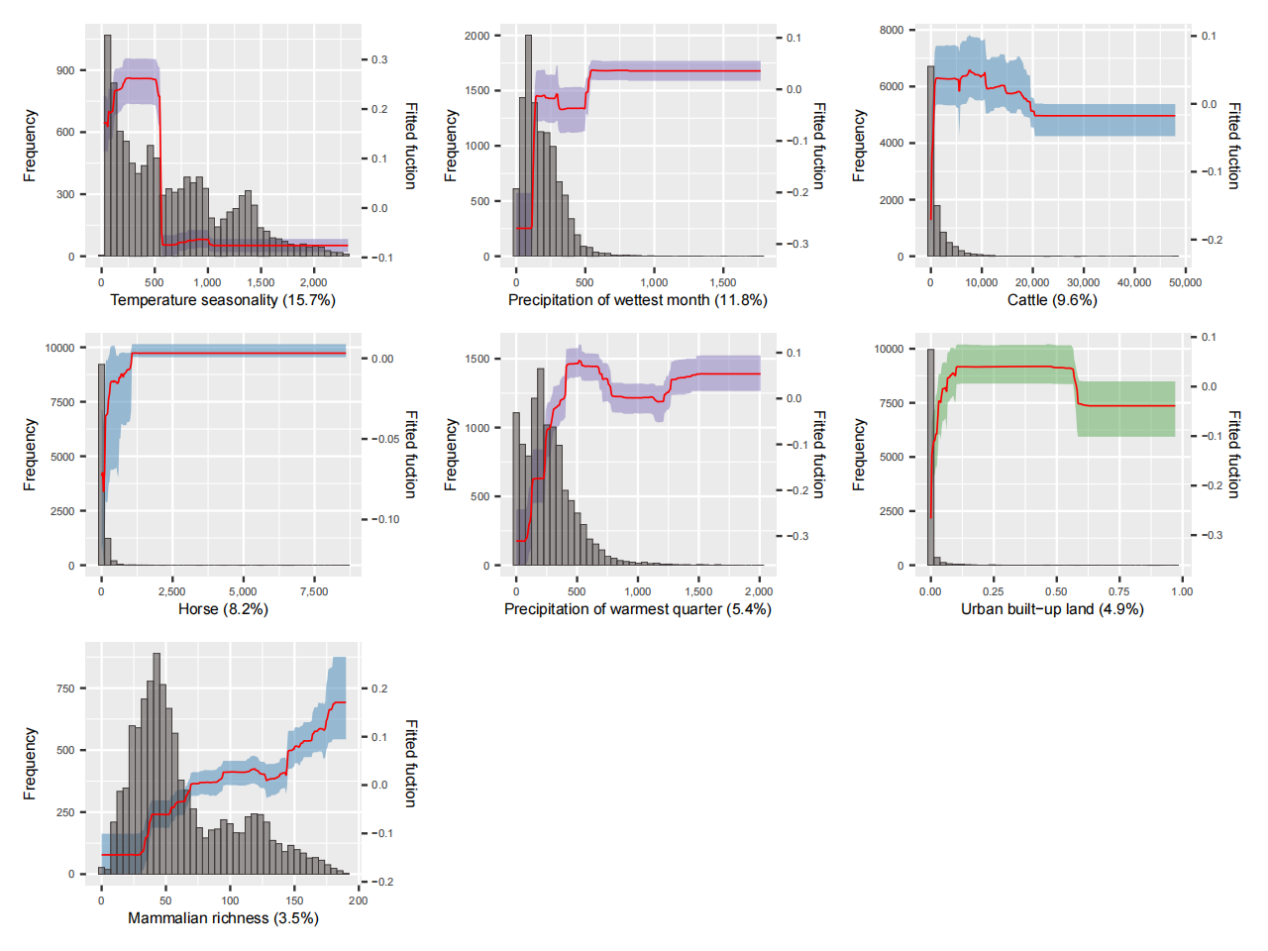


Figure S4: The recorded and predicted distributions of *Dermacentor reticulatus* in Europe. (A) Recorded locations of *Dermacentor reticulatus*. (B) Predicted HSI of *Dermacentor reticulatus* based on the BRT model. (C) Predicted HSI and relative uncertainty into four segments by their corresponding 80th, 90th and 95th percentiles, respectively, with the colors from light to deep representing the values from low to high.


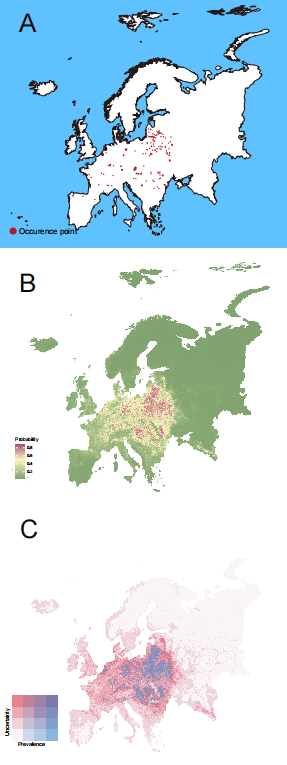


Figure S5: The recorded and predicted distributions of *Rhipicephalus microplus* within global range. (A) Recorded locations of *Rhipicephalus microplus*. (B) Predicted HSI of *Rhipicephalus microplus* based on the BRT model. (C) Predicted HSI and relative uncertainty into four segments by their corresponding 80th, 90th and 95th percentiles, respectively, with the colors from light to deep representing the values from low to high.

**
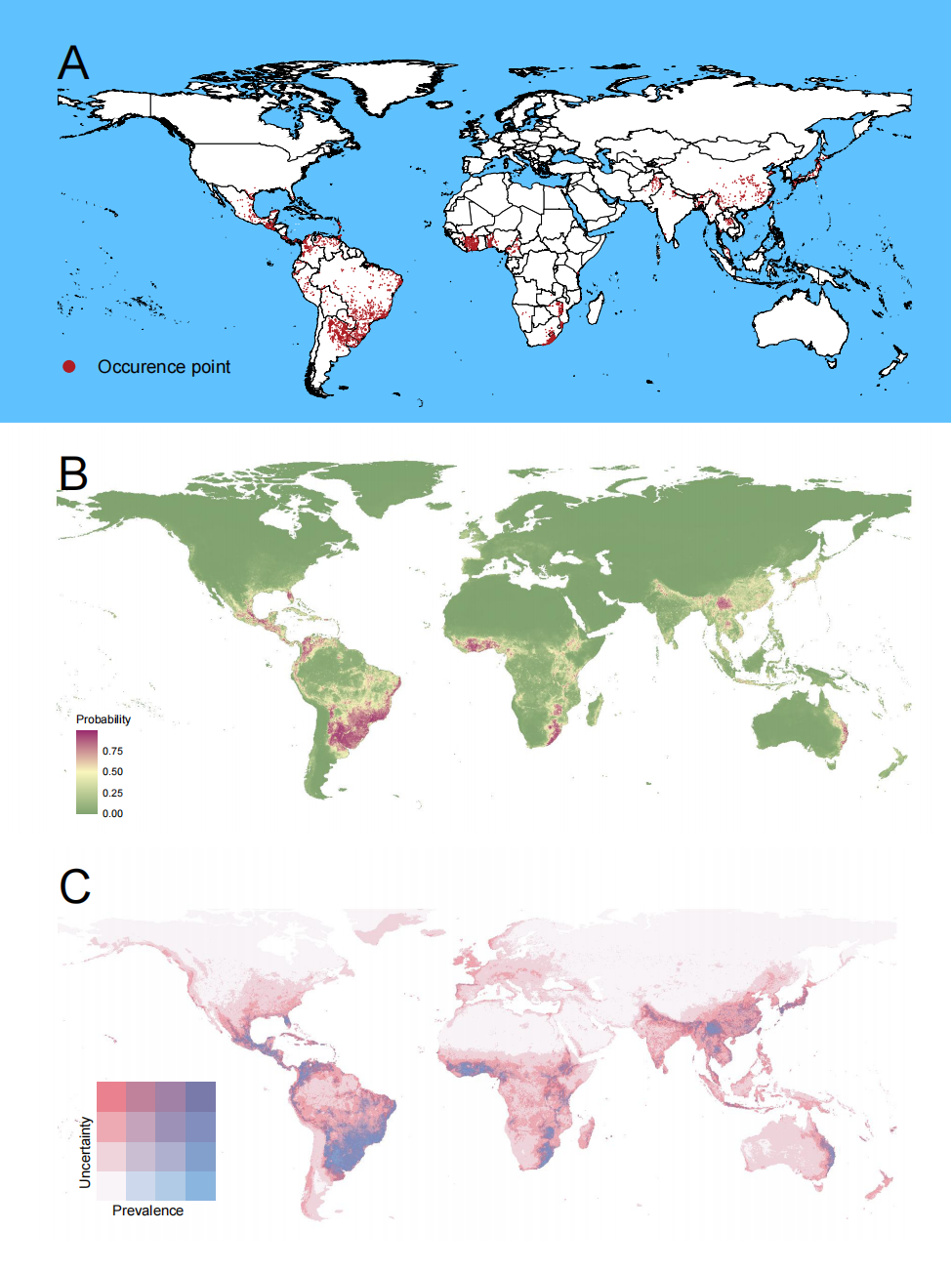
**

Figure S6: Predictive performance of the three machine-learning algorithms. ROC curves and AUC values of the BRT (left, red), RF (middle, blue) and LASSO regression (right, orange) over 100 models are shown. Roman numerals 1-6 correspond to the six major *Babesia* species: (I) *Babesia microti*; (II) *Babesia* sp. venatorum; (III) *Babesia divergens*; (IV) *Babesia bigemina;* (V)*Babesia bovis*; (VI)*Babesia odocoilei*.

**
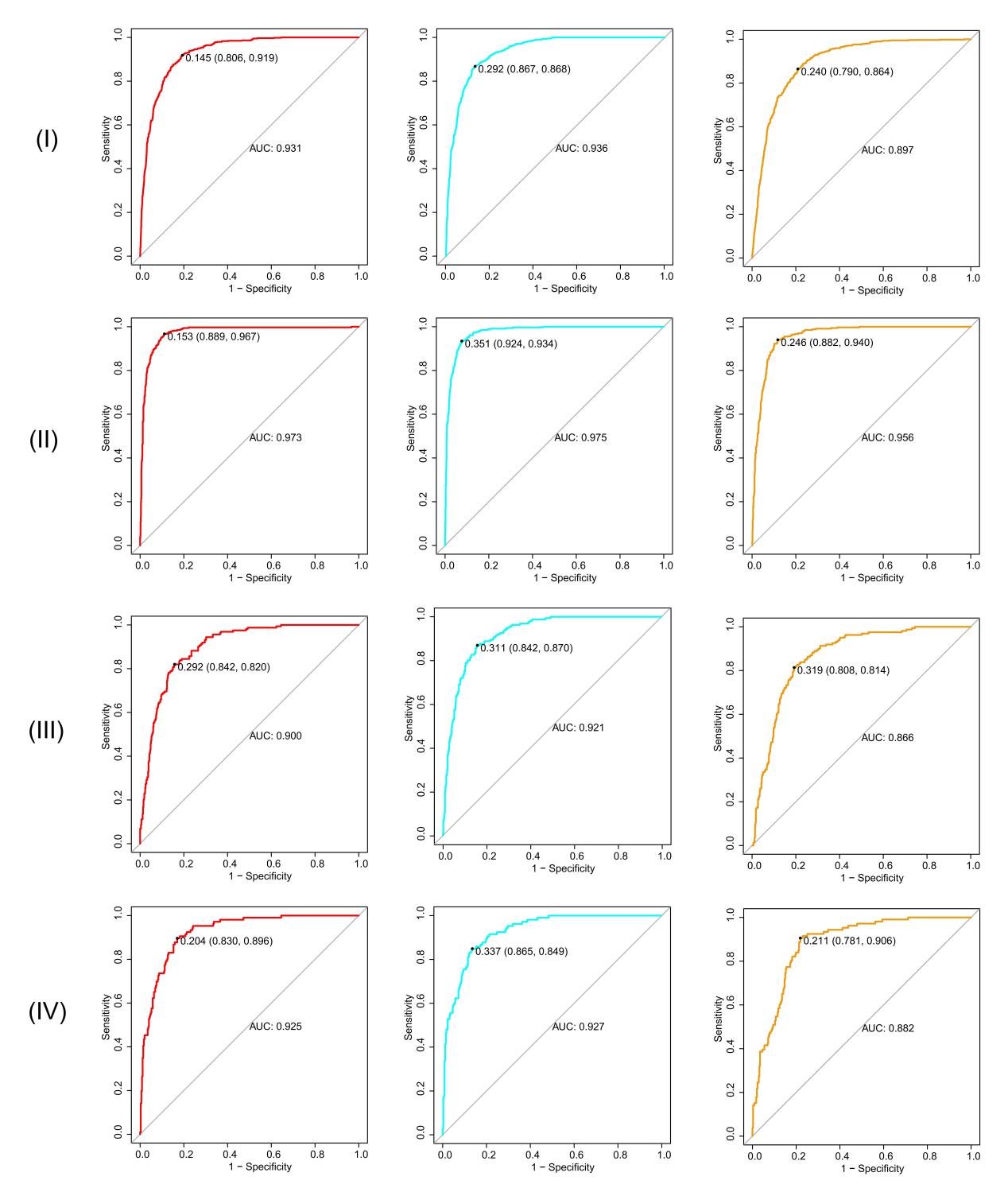
**

**
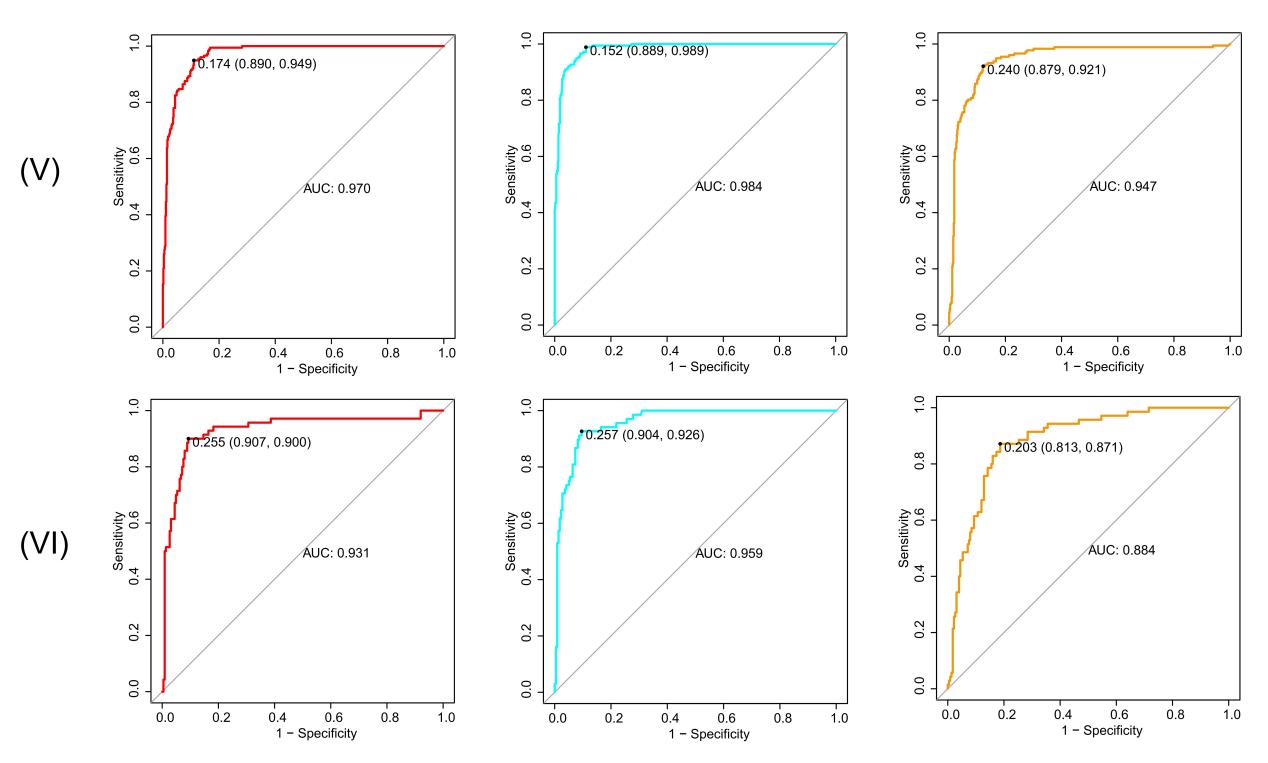
**

Figure S7: Effects of major predictors (RCs >5%) for presence of *Babesia microti* based on RF models. The mean curves (red) and 95% percentiles (blue, biological variables; purple, ecoclimatic variables; green, environmental variables; red, socioeconomic variables) show the predicted probability of occurrence. The histograms show the frequency distributions of the predictors.


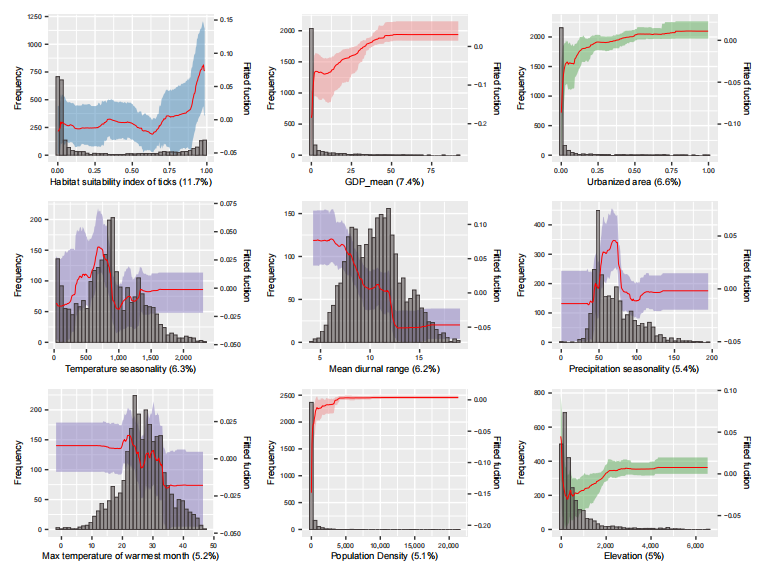


Figure S8: Effects of major predictors (RCs >5%) for presence of *Babesia* sp. venatorum based on RF models. The mean curves (red) and 95% percentiles (blue, biological variables; purple, ecoclimatic variables; green, environmental variables; red, socioeconomic variables) show the predicted probability of occurrence. The histograms show the frequency distributions of the predictors.


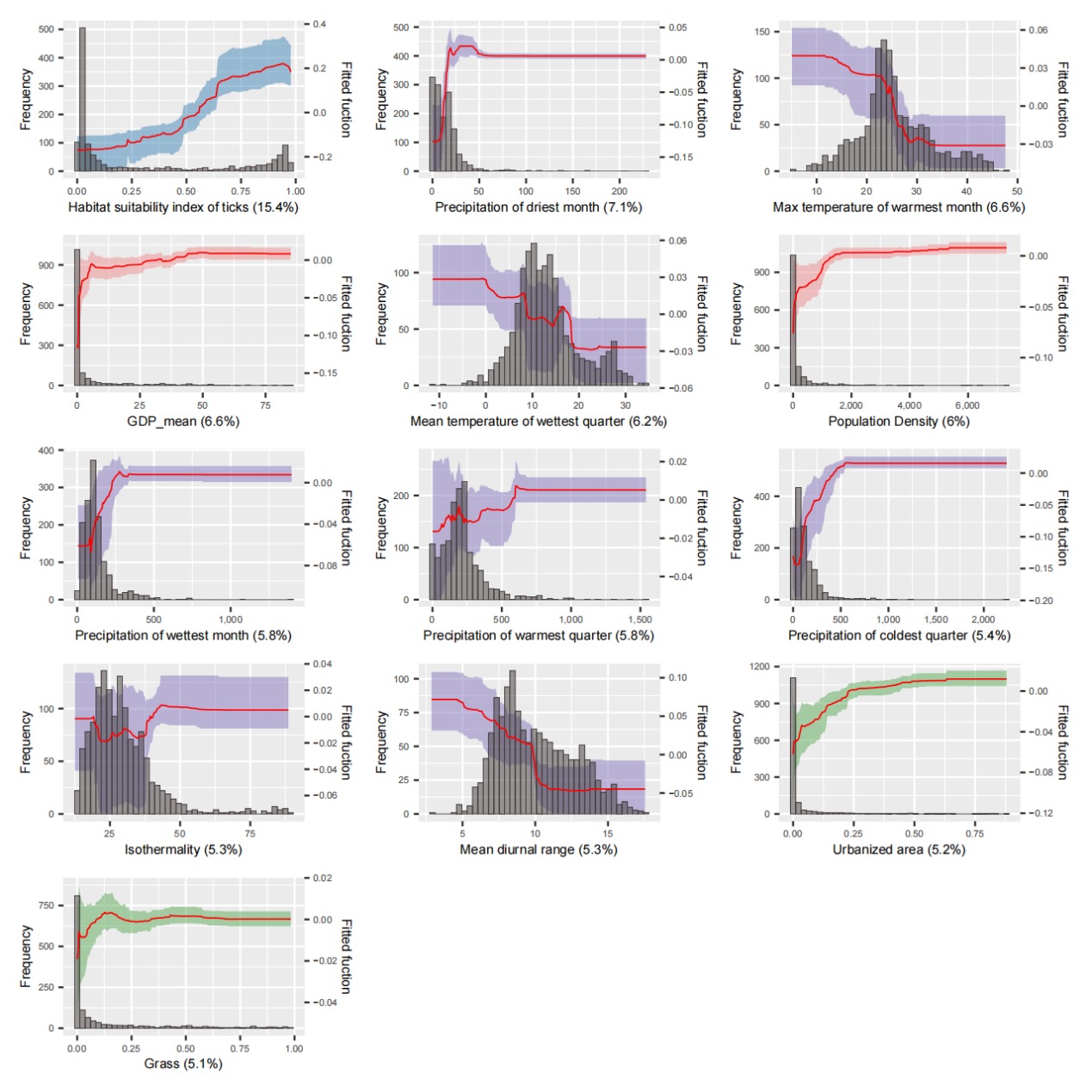


Figure S9: Effects of major predictors (RCs >5%) for presence of *Babesia* *divergens* based on RF models. The mean curves (red) and 95% percentiles (blue, biological variables; purple, ecoclimatic variables; green, environmental variables; red, socioeconomic variables) show the predicted probability of occurrence. The histograms show the frequency distributions of the predictors.


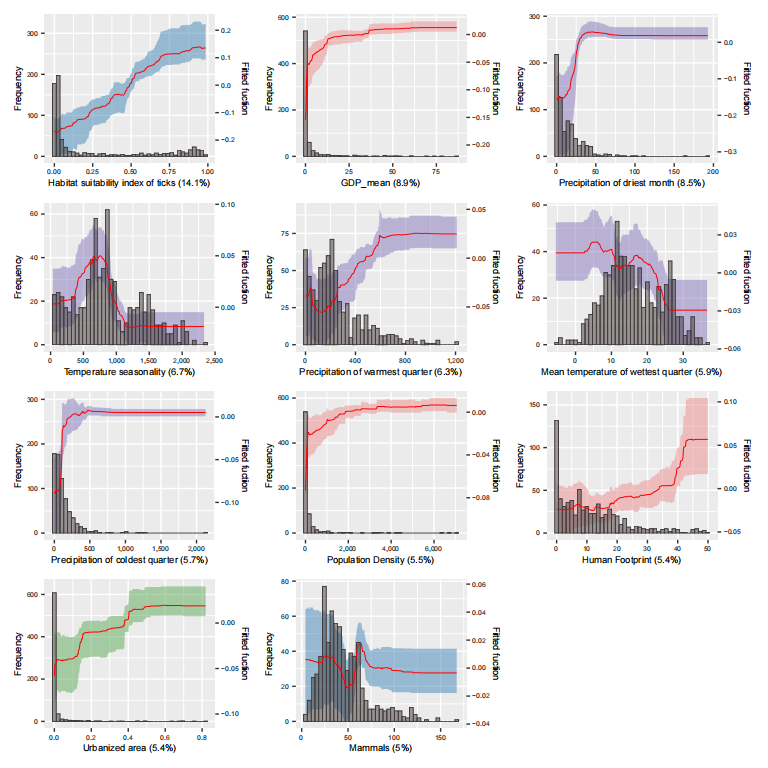


Figure S10: Effects of major predictors (RCs >5%) for presence of *Babesia bigemina* based on RF models. The mean curves (red) and 95% percentiles (blue, biological variables; purple, ecoclimatic variables; green, environmental variables; red, socioeconomic variables) show the predicted probability of occurrence. The histograms show the frequency distributions of the predictors.


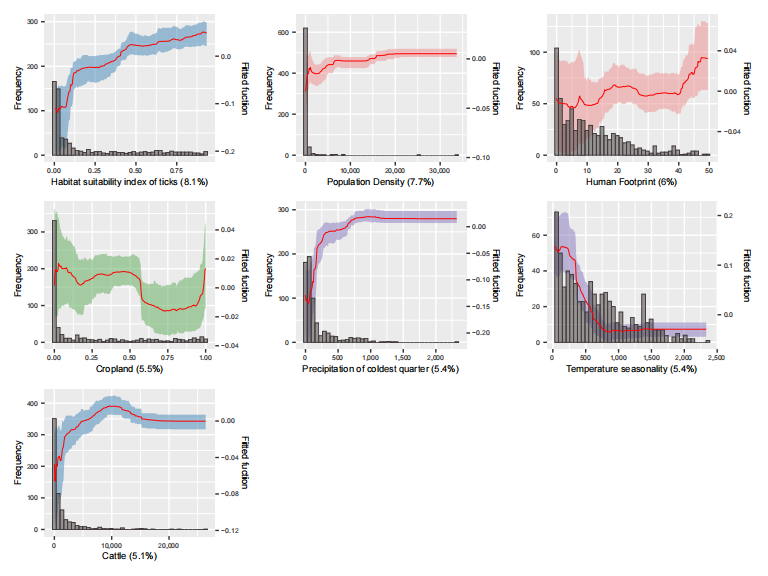


Figure S11: Effects of major predictors (RCs >5%) for presence of *Babesia bovis* based on RF models. The mean curves (red) and 95% percentiles (blue, biological variables; purple, ecoclimatic variables; green, environmental variables; red, socioeconomic variables) show the predicted probability of occurrence. The histograms show the frequency distributions of the predictors.


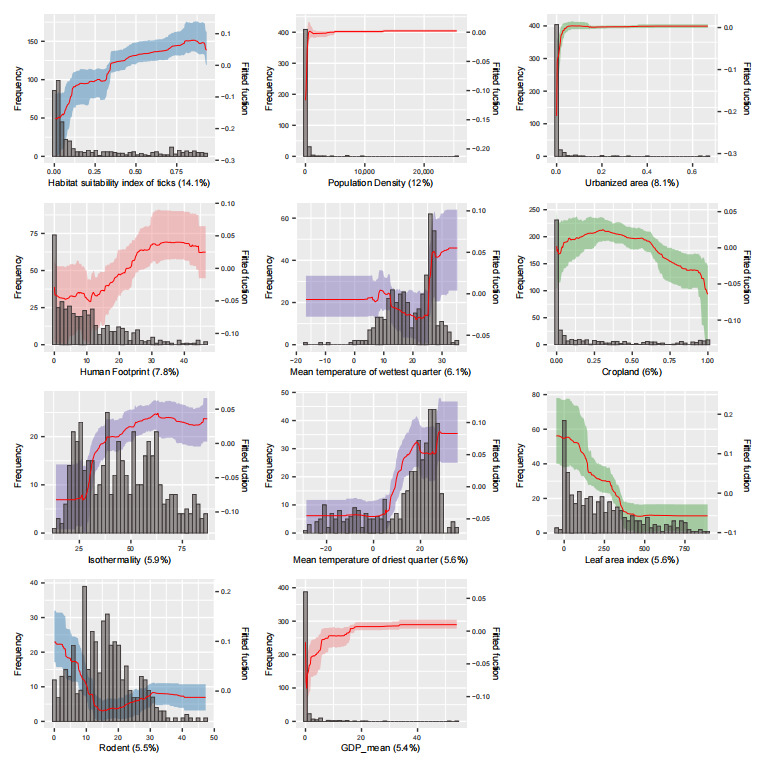


Figure S12: Effects of major predictors (RCs >5%) for presence of *Babesia odocoilei* based on RF models. The mean curves (red) and 95% percentiles (blue, biological variables; purple, ecoclimatic variables; green, environmental variables; red, socioeconomic variables) show the predicted probability of occurrence. The histograms show the frequency distributions of the predictors.


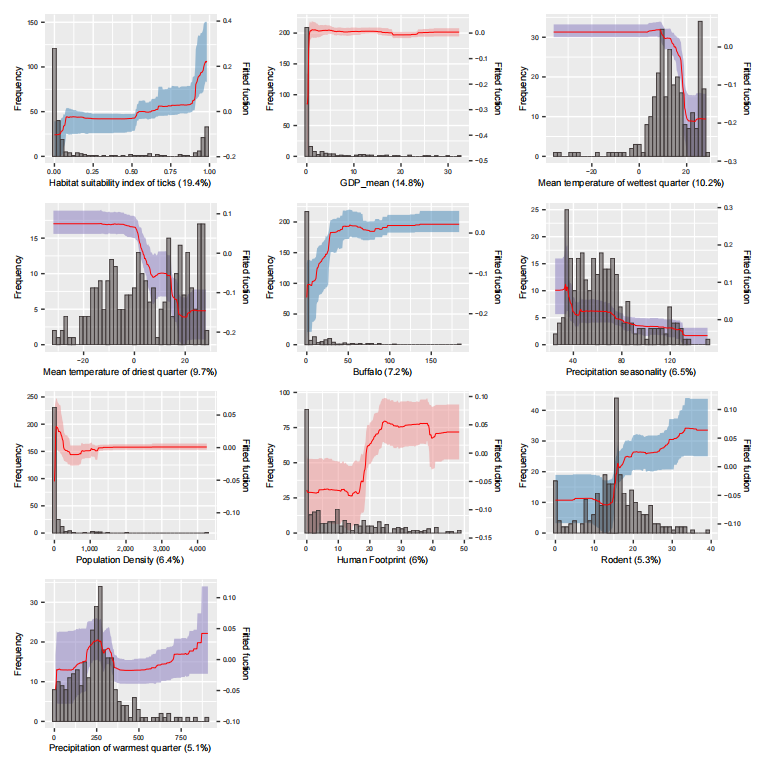


Figure S13: The recorded and predicted distributions of *Babesia microti* within global range. (A) Recorded locations of *Babesia microti* detected from arthropod vectors, animals and humans. (B) Predicted risk probability of *Babesia microti* based on the random forest model.


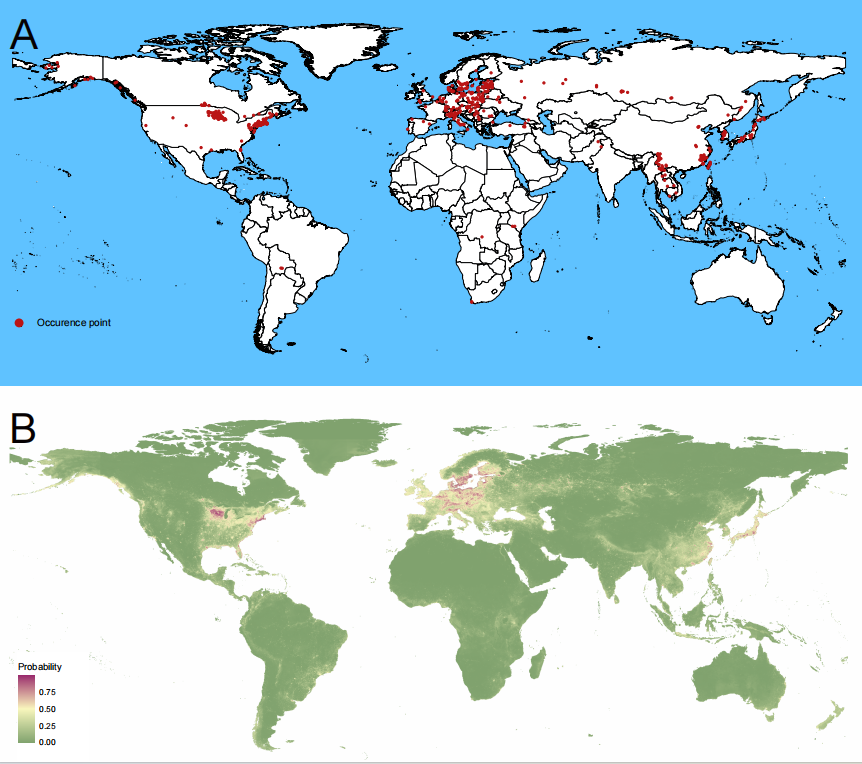


Figure S14: The recorded and predicted distributions of *Babesia* sp. venatorum in Eurasia. (A) Recorded locations of *Babesia* sp. venatorum detected from arthropod vectors, animals and humans. (B) Predicted risk probability of *Babesia* sp. venatorum based on the random forest model.


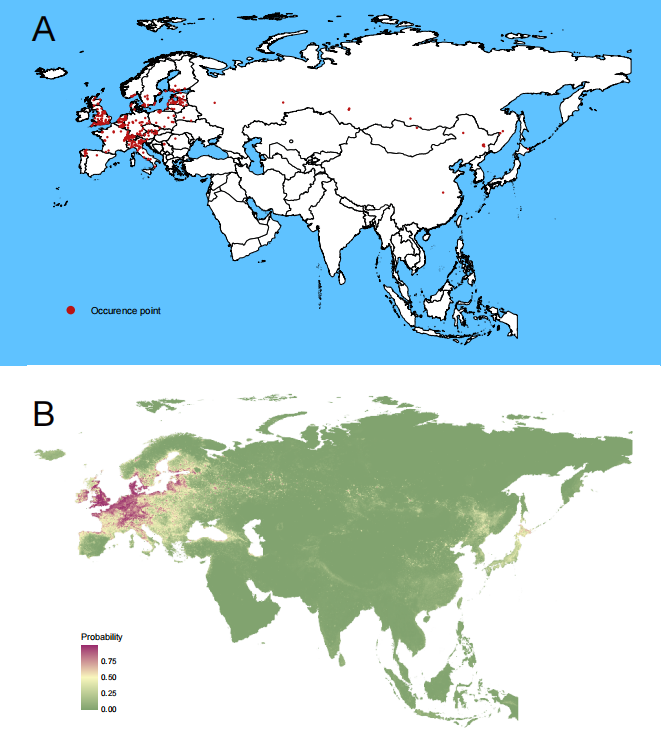


Figure S15: The recorded and predicted distributions of *Babesia divergens* in Eurasia and Africa. (A) Recorded locations of *Babesia divergens* detected from arthropod vectors, animals and humans. (B) Predicted risk probability of *Babesia divergens* based on the random forest model.
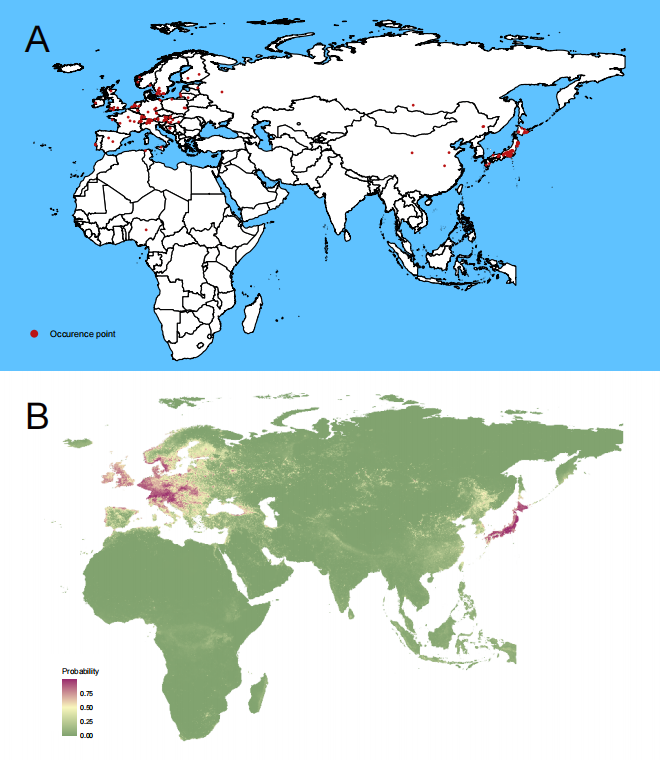


Figure S16: The recorded and predicted distributions of *Babesia bigemina* within global range. (A) Recorded locations of *Babesia bigemina* detected from arthropod vectors, animals and humans. (B) Predicted risk probability of *Babesia bigemina* based on the random forest model.
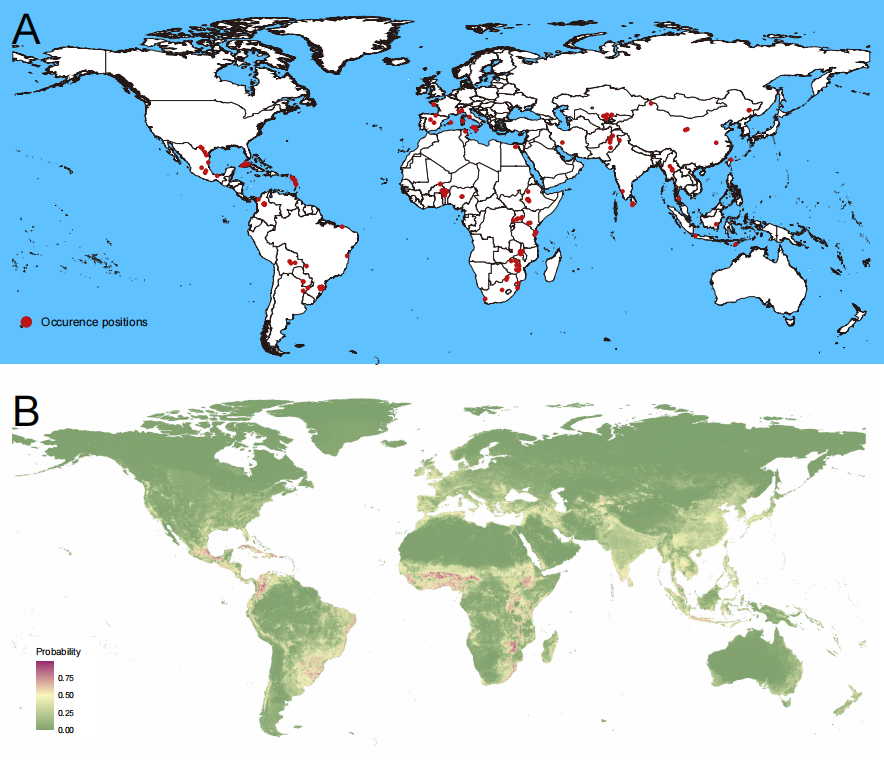


Figure S17: The recorded and predicted distributions of *Babesia bovis* within global range. (A) Recorded locations of *Babesia bovis* detected from arthropod vectors, animals and humans. (B) Predicted risk probability of *Babesia bovis* based on the random forest model.
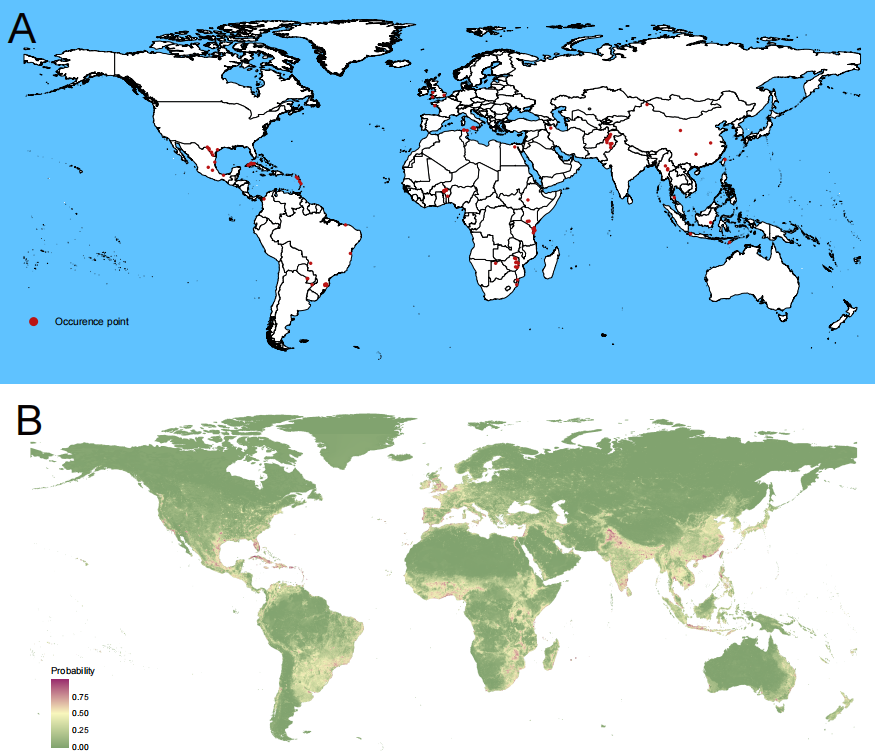


Figure S18: The recorded and predicted distributions of *Babesia odocoilei* in Americas. (A) Recorded locations of *Babesia odocoilei* detected from arthropod vectors, animals and humans. (B) Predicted risk probability of *Babesia odocoilei* based on the random forest model.


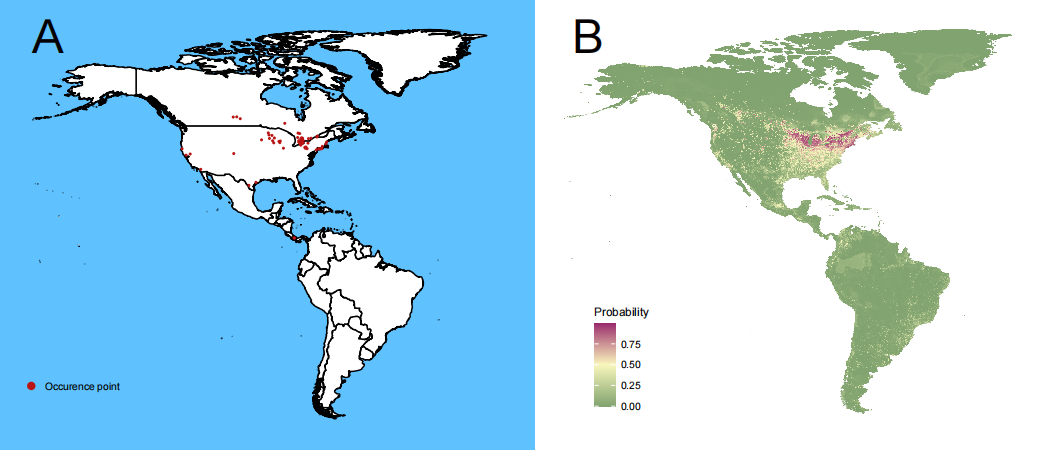

Supplement: Supporting Information 1 — Table S1: the inclusion and exclusion criteria of screening publications. Table S2: the laboratory tests used to detect Babesia infections in the reviewed studies. Table S3: list of variables extracted from reviewed studies. Table S4: the references for all the Babesia species. Table S5: the spatial resolution, study duration, and source of the included data. Table S6: ecological factors potentially associated with the ticks and tick pathogens used in the modeling analysis. Table S7: variables used for ecological modeling in this study. Table S8: the number of studies and positive occurrence grids for the ten Babesia species with reports of confirmed human infection. Table S9: the tick species carrying the six major Babeisa species. Table S10: the number of studies and occurrence locations for the two tick species. Table S11: vectors with evidence of biting human. Table S12: the coinfection of Babesia species and their infected vectors. Table S13: the number of human cases of Babesia infection. Table S14: clinical characteristics of human infections with Babesia species. Table S15: the RCs of significant contributors to the occurrence of two main species of ticks based on BRT models. Table S16: the VIF values for each variable in the models of Babesia. Table S17: comparison of AUC values for the models of Babesia species using two area thresholds. Table S18: comparison of AUC values for the models of Babesia species using two sampling methods. Table S19: the RCs of significant contributors to the spatial distribution of the six major Babesia species based on RF models. Figure S1: the distributions of other Babesia species. Figure S2: effects of major predictors (RCs >3%) for presence of Dermacentor reticulatus based on BRT models. Figure S3: effects of major predictors (RCs >3%) for presence of Rhipicephalus microplus based on BRT models. Figure S4: the recorded and predicted distributions of Dermacentor reticulatus in Europe. Figure S5: the recorded and predicted d [file 5889219.f1.docx]
