# Supplementary material for: Mapping the Global Distribution of Babesia Infections
Source: Transbound Emerg Dis. 2025 Nov 24;2025:5889219. doi: 10.1155/tbed/5889219 (PMC12668841; doi:10.1155/tbed/5889219)
Supplement: Supporting Information 3 — Supplementary methods and results. [file 5889219.f3.docx]

**Supplementary Methods and Results**

# Supplementary Methods

*Data extraction*

From each included study, we extracted the following data: article title, publication date, sample collection date, sample collection location, geographical coordinates of the location, *Babesia* species, detection method, vector species/animal species/human, total number of samples tested, number of positive results, symptoms of confirmed human cases when infected with the identified *Babesia* species and without other pathogen co-infections. The laboratory confirmed infections were limited to those determined by microscopy, molecular assay or pathogen isolation evidence.

*Assembling occurrence data and covariates*

We created a global grid-map with a resolution of 10 × 10 km using ArcGIS 10·7 (Esri Inc, Redlands, CA, USA) and then associated each grid with ecological variables. Each occurrence was matched to a grid on a map based on its coordinates. For occurrence records of polygon type, the grid that contained the centroid of the polygon was designated as the occurrence grid. If multiple records were associated with the same grid, the occurrence was counted only once. For ecological modeling, ecological variables needed to be associated with each grid (whether an occurrence or non-occurrence). The average value of each ecological variable within each grid was calculated over the corresponding time span [1,2].To minimize potential ecological fallacy, we first excluded all huge polygon occurrence records from ecological modelling due to insufficient resolution [3]. For polygon occurrence records with an area no larger than 400 km^2^, we calculated the mean of each ecological variable across all grids within the polygon and associated the mean value with the occurrence grids, that was, the grids containing the centroid of the polygon. We compiled data on 44 potential ecological variables that could be associated with *Babesia* occurrences (Table S6) [1,2,4,5]. These variables were categorized into four groups: 19 ecological climate variables, 15 environmental variables, seven biological variables, and three socio-economic variables. All variables were processed in a grid map with a resolution of 10 km. Ecological climate, environmental, and biological variables have been widely used to predict tick distribution and the risk of tick-borne diseases. The increasing frequency of tick-borne disease occurrence is also associated with complex socio-economic factors, which may expand the interface between humans, animals, ticks, and the environment and impact the discovery and monitoring of pathogens [6,7].

*Niche modelling of the main tick species*

First, we extracted all records of the six main Babesia-infected ticks from studies where the total number of tick detections exceeded 100. We conducted a meta-analysis to obtain the positivity rate of infection for each *Babesia* species. We calculated the pooled effect size for the positivity rate of each *Babesia* species carried by each tick species using both random-effects and fixed-effects models. Considering the heterogeneity among studies, we used the random-effects model when significant heterogeneity among studies was observed, and the fixed-effects model when heterogeneity was not significant. The significance of heterogeneity was determined using the I² statistic, where heterogeneity was considered significant when the I² statistic exceeded 75%, and nonsignificant otherwise. Then we excluded tick species with less than four literatures to reduce random errors. Finally, we selected the tick species with the highest number of literature and the tick species with an infection rate greater than half of the infection rate of the former species. We identified five predominant tick species, namely, *Ixodes persulcatus*, *Ixodes ricinus*, *Ixodes scapularis, Dermacentor reticulatus* and *Rhipicephalus microplus*. The model predictions for the first three tick species are derived from previous studies [2]; hence, we only conducted modeling analysis for the last two tick species. We performed a systematic search of PubMed and Web of Science for published studies or reports on *D. reticulatus* and *R. microplus* without any language restrictions from January 1980 to December 2022. Search results were exported through EndNote (version 19.1), duplicates were removed.

The spatial data associated with tick collection was obtained for those that met the following criteria: (I) study on pathogens in ticks providing locations of sampling; (II) confirmation of tick species with molecular evidence or identification of morphology; (III) providing accurate locations of tick collection or geographic distribution figures. All data that did not provide precise coordinates were excluded from the ecological niche modeling. The location information for each tick species was also retrieved from the database of the Global Biodiversity Information Facility (GBIF) and VectorMap. The accuracy of the coordinates was checked through Google Earth, and some samples with apparent errors in their geographic location coordinates were removed. After the above screening process, the geographical coordinates of each tick species were assembled and overlapped on a global grid of 10 × 10 km pixels, and only one code of grid was retained.

Based on the distribution information of tick species, *R. microplus* was distributed across multiple continents. *D. reticulatus* occurred in the Europe. The habitat suitability index of *R. microplus* would be used as a predictor for the niche modelling of *Babesia bigemina* and *Babesia bovis*. *I. ricinus* would be used for niche modelling of *Babesia odocoilei*. *I. persulcatus* and *I. ricinus* would be combined for niche modelling of *Babesia divergens* and *Babesia* sp. venatorum. *I. scapularis*, *D. reticulatus*, *I. persulcatus*, and *I. ricinus* would be combined for niche modelling of *Babesia microti*. Multiple ticks were integrated as follows: (I) in the region where the four tick species had not been reported, the value was set to zero; (II) otherwise, the value was the maximum of the four ticks habitat suitability indexes.

All occurrence grids of each tick species were considered as “cases”. For each occurrence grids, we sampled the pseudo-absence grids as “controls” with a control-to-case ratio of 3:1 for the subsequent modelling. The sampling was restricted to grids within the study area that were more than 30 km away from “case” grids with the method as follows: (1) randomly sampled 10000 grids from the sampling area as the candidate “controls”; (2) randomly sampled the pseudo-absence locations from the candidate “controls” at a ratio of 3:1 around the occurrence locations [5,8,9]. The boosted regression trees (BRT) model was used to calculate the habitat suitability index for each of the seven main tick species in the corresponding occurrence areas, with the selected 41 variables excepting socioeconomic variables as candidate predictors (Table S7).

To confirm the accuracy of the tick niche modelling, we performed internal validation, which evaluated the robustness of the model itself by calculating the relative uncertainty of the predicted distribution for each tick species. The relative uncertainty for each grid was computed as the ratio of the 95% uncertainty intervals to the predicted suitability.

*Niche modelling of six major Babesia species*

To explore the relationship between the risk of *Babesia* occurrences and ecoclimatic, environmental, biological, and socioeconomic variables, three machine-learning models, including BRT, random forest (RF) and least absolute shrinkage and selection operator (LASSO) logistic regression, were performed and compared to obtain the best predictive performance. The pseudo-absence locations were randomly sampled around the locations of *Babesia* events which were considered as “cases”, with a range of all the grids except for the grids within 30 km around the “cases” using the method as follows: (I) For globally distributed *Babesia* species, including *Babesia microti*, *Babesia bigemina* and *Babesia bovis*, pseudo-absence locations were sampled randomly within a range of 30–3000 km around the occurrence locations with a 3:1 ratio ; (II) The remaining three *Babesia* species, pseudo-absence locations were sampled randomly with a 3:1 ratio, without setting a maximum range for their distribution on continents. To avoid overfitting and to improve interpretability of the models, we first screened for multicollinearity among candidate predictors. We used the R package 'usdm' to calculate the variance inflation factor (VIF) and exclude variables with a VIF greater than 10 (Table S16). We then fitted an initial model for each species, and predictors with relative contributions (RCs) greater than 3% were retained for the formal modelbuilding [1,2]. We then sampled 80% of the training set and 20% of the test set via random splitting and fitted the three models separately, which was repeated 100 times. The machine learning model with the largest average area under the curve (AUC) over 100 repeats was selected to calculate the relative contribution (RCs) of the major predictors and make a final prediction for the risk of *Babesia* occurrence. All remaining variables were fitted in the three models for niche modelling, but only those variables with an average RC greater than 5% were shown. The optimal threshold value used for the final prediction of the presence or absence of the *Babesia* species in the study region was based on the Youden index derived from the average AUC over the 100 models. To minimize potential ecological fallacy, we must exclude large polygon occurrence records from ecological modelling using an area cutoff. To minimize potential ecological fallacy, we excluded records with larger areas from ecological modelling by using an area cutoff. We used 400 km² as thresholds and the polygon occurrence records exceeding these thresholds were excluded from ecological modelling. And a higher area cutoff might make the average conditions in some areas unable to reasonably reflect conditions where the observation was actually made. Therefore, we have constructed a comparison model with another threshold of 100 km². For polygon occurrence records, the center grids were considered as the cases, while the other grids within the polygon were excluded from the sampling range of the “controls”. Meanwhile, the average of all the grids within the polygon were taken as the values of various predictors at the center grids.

To verify the effect of different sampling methods on the modelling results, we obtained geographical data on SFG rickettsiae, relapsing fever group Borrelia, tick-borne encephalitis virus, and Anaplasmataceae. The 5 089 grids in which these point data were located served as the candidate "controls" for each *Babesia* species within the study area. The sampling was restricted to grids within the study area that were more than 30 km away from “case” grids with the method as follows: randomly sampled the pseudo-absence locations from the candidate “controls” at different ratio range of three times around the occurrence locations. The modelling scheme remained the same.

(1) BRT model

The hyperparameters of the BRT model were set by grid search to obtain the best-fit effect. In order to avoid underestimation of error and inappropriate selection of models, we implemented a 10-fold block cross-validation approach for the niche modelling using the package “blockCV” [10]. We compared the mean residuals of the spatial autocorrelation for different block sizes and determined 300 km as the optimal value for the block. The modelling dataset was divided into different blocks in the raster map by spatial autocorrelation and then randomly encoded, with each raster within a block assigned a unique code. The train and test datasets were segmented by these codes, and the optimal modelling parameters were determined by the best AUC obtained from 10-fold block cross-validation. We then fitted an initial model for each tick (or *Babesia*) species to obtain the best number of tree and apply in the subsequent modelling. In the model, we randomly divided the data into an 80% training set and a 20% test set and fitted a BRT model, which was repeated 100 times to obtain 100 models based on the 100 resampled training sets for each target tick (or *Babesia*) species [1,2,5,11]. Using these presence and pseudo-absence locations and ecological predictors, BRT models were fitted using the “gbm.step” function in “dismo” package in R.

(2) Random forest

Random forest (RF) is another classical ensemble learning model widely used. The training algorithm for RF is based on bootstrap aggregating. Each tree is trained on many bootstrap samples and then evaluated using the remaining data to produce more accurate classifications. The unknown class of an observation will be calculated by the majority vote of the out-of-bag predictions for that observation. We optimized the hyperparameters by grid search with a 10-fold block cross-validation process to avoid inappropriate model selection. The hyperparameter selection process was the same as in BRT. The R packages “randomForest” were used to develop the random forest model.

(3) LASSO regression

We used L1-penalized least absolute shrinkage and selection regression for multivariate analysis, augmented with 10-fold block cross validation for internal validation. This is a logistic regression model that penalizes the absolute size of the coefficients, where the sum of absolute values of coefficients is multiplied by a weight coefficient λ and then added to the traditional loss function. With larger penalties, the estimates of the weaker factors shrink towards zero, so that only the strongest predictors remain in the model. The optimal λ was chosen via 10-fold block cross validation to minimize the average misclassification error. Subsequently, variables identified by least absolute shrinkage and selection operator (LASSO) regression analysis were entered into traditional logistic regression models without penalty (as there is no predictor of more interest than others, double selection was not performed) [12]. The package “glmnet” in R was used to perform the LASSO regression, and optimal λ was chosen using the cv. glmnet function.

(4) Model evaluation

Similar to the BRT, we obtained 100 models as a model assembly for RF and LASSO as well as by randomly splitting the data into training and test sets. The RCs of all predictors and the area under the curves (AUCs) for test sets were averaged over the 100 models in the assembly to represent the final estimation results and predictive performance of the model assembly. We selected the best algorithm in terms of the highest average test AUC to map the global distribution of *Babesia* species. To determine model-predicted high-risk areas for each *Babesia* species, we chose a cut-off value that maximizes sensitivity specificity along the average receiver operating characteristic (ROC) curve of the model assembly of the chosen algorithm [13,14]. Grids with an average predicted probability (over the 100 models) above the cut-off value were considered as having a high risk of presence of the corresponding *Babesia* species. For each species, the area and population size of the model-predicted high-risk areas were calculated.

# Supplementary Results

*Symptom characteristics of babesiosis*

After excluding patients with concurrent infections with other pathogens and those with fewer than 10 cases, we extracted clinical symptom data from 691 patients infected with three species of *Babesia* parasites. The clinical cases reported for *Babesia microti* were the highest, with 622 patients accounting for 90·0% of the total reported cases. Influenza-like symptoms were most common, primarily fever, fatigue, chills, and sweats; Next were gastrointestinal symptoms, with decreased appetite and nausea occurring more frequently; Musculoskeletal symptoms were less common, such as myalgia and arthralgia.

*Validation of tick niche modelling*

The relationship between relative uncertainty and the habitat suitability for each grid on tick species was mapped, indicating that the predicted results are more robust in the areas with higher HSI (Supplementary Fig. 4-5). Moreover, in agreement with the study by Perez-Martinez MB et al.,^15^ the areas with high HSI for *R. microplus* are concentrated in Latin America and southern Africa, along with parts of southern Asia and a small portion of Australia.

*Ecological associations of Babesia occurrences*

The HSI of vector ticks was the most significant factor contributing to the *Babesia* occurrences. Higher GDP and greater population density were associated with a RC of ≥5% for five *Babesia* species, except for *Babesia bigemina*. Higher human footprint was more suitable for the survival of *Babesia divergens*, *Babesia bigemina*, *Babesia bovis* and *Babesia odocoilei*. More than four climate factors significantly affected the presence of *Babesia* sp. venatorum and *Babesia divergens*. Increased density of cattle was negatively associated with the distribution of *Babesia bigemina*, which was the opposite of the relationship between *Babesia bovis* and rodent richness. However, higher density of cattle and rodent richness were more suitable for the survival of *Babesia odocoilei.*

**Supplementary references**

[1] Y. Y. Zhang, Y. Q. Sun, J. J. Chen, A. Y. Teng, T. Wang, H. Li, S. I. Hay, L. Q. Fang, Y. Yang, & W. Liu. "Mapping the global distribution of spotted fever group rickettsiae: a systematic review with modelling analysis." *Lancet Digit Health* 5, no. 1 (2023): e5-e15.

[2] T. Tang, Y. Zhu, Y. Y. Zhang, J. J. Chen, J. B. Tian, Q. Xu, B. G. Jiang, G. L. Wang, N. Golding, M. L. Mehlman, C. L. Lv, S. I. Hay, L. Q. Fang, & W. Liu. "The global distribution and the risk prediction of relapsing fever group Borrelia: a data review with modelling analysis." *Lancet Microbe* 5, no. 5 (2024): e442-e451.

[3] J. P. Messina, D. M. Pigott, N. Golding, K. A. Duda, J. S. Brownstein, D. J. Weiss, H. Gibson, T. P. Robinson, M. Gilbert, G. R. William Wint, P. A. Nuttall, P. W. Gething, M. F. Myers, D. B. George, & S. I. Hay. "The global distribution of Crimean-Congo hemorrhagic fever." *Trans R Soc Trop Med Hyg* 109, no. 8 (2015): 503-513.

[4] T. Allen, K. A. Murray, C. Zambrana-Torrelio, S. S. Morse, C. Rondinini, M. Di Marco, N. Breit, K. J. Olival, & P. Daszak. "Global hotspots and correlates of emerging zoonotic diseases." *Nat Commun* 8, no. 1 (2017): 1124.

[5] G. P. Zhao, Y. X. Wang, Z. W. Fan, Y. Ji, M. J. Liu, W. H. Zhang, X. L. Li, S. X. Zhou, H. Li, S. Liang, W. Liu, Y. Yang, & L. Q. Fang. "Mapping ticks and tick-borne pathogens in China." *Nat Commun* 12, no. 1 (2021): 1075.

[6] D. Miao, K. Dai, G. P. Zhao, X. L. Li, W. Q. Shi, J. S. Zhang, Y. Yang, W. Liu, & L. Q. Fang. "Mapping the global potential transmission hotspots for severe fever with thrombocytopenia syndrome by machine learning methods." *Emerg Microbes Infect* 9, no. 1 (2020): 817-826.

[7] A. R. Magalhães, C. T. Codeço, J. C. Svenning, L. E. Escobar, P. Van de Vuurst, & T. Gonçalves-Souza. "Neglected tropical diseases risk correlates with poverty and early ecosystem destruction." *Infect Dis Poverty* 12, no. 1 (2023): 32.

[8] J. VanDerWal, L. P. Shoo, C. Graham, & S. E. Williams. "Selecting pseudo-absence data for presence-only distribution modeling: How far should you stray from what you know?" *Ecological Modelling* 220, no. 4 (2009): 589-594.

[9] M. Barbet-Massin, F. Jiguet, C. H. Albert, W. J. M. i. E. Thuiller, & Evolution. "Selecting pseudo‐absences for species distribution models: how, where and how many?" 3 (2012).

[10] R. Valavi, J. Elith, J. Lahoz-Monfort, & G. Guillera-Arroita. "blockCV: an R package for generating spatially or environmentally separated folds for k-fold cross-validation of species distribution models." *Methods in Ecology and Evolution* 10 (2018).

[11] T. Wang, Z. W. Fan, Y. Ji, J. J. Chen, G. P. Zhao, W. H. Zhang, H. Y. Zhang, B. G. Jiang, Q. Xu, C. L. Lv, X. A. Zhang, H. Li, Y. Yang, L. Q. Fang, & W. Liu. "Mapping the Distributions of Mosquitoes and Mosquito-Borne Arboviruses in China." *Viruses* 14, no. 4 (2022).

[12] W. Liang, H. Liang, L. Ou, B. Chen, A. Chen, C. Li, Y. Li, W. Guan, L. Sang, J. Lu, Y. Xu, G. Chen, H. Guo, J. Guo, Z. Chen, Y. Zhao, S. Li, N. Zhang, N. Zhong, & J. He. "Development and Validation of a Clinical Risk Score to Predict the Occurrence of Critical Illness in Hospitalized Patients With COVID-19." *JAMA Intern Med* 180, no. 8 (2020): 1081-1089.

[13] E. F. Schisterman, N. J. Perkins, A. Liu, & H. Bondell. "Optimal cut-point and its corresponding Youden Index to discriminate individuals using pooled blood samples." *Epidemiology* 16, no. 1 (2005): 73-81.

[14] M. D. Ruopp, N. J. Perkins, B. W. Whitcomb, & E. F. Schisterman. "Youden Index and optimal cut-point estimated from observations affected by a lower limit of detection." *Biom J* 50, no. 3 (2008): 419-430.

[15] M. B. Perez-Martinez, D. A. Moo-Llanes, C. N. Ibarra-Cerdeña, D. Romero-Salas, A. Cruz-Romero, K. M. López-Hernández, & M. Aguilar-Dominguez. "Worldwide comparison between the potential distribution of Rhipicephalus microplus (Acari: Ixodidae) under climate change scenarios." *Med Vet Entomol* 37, no. 4 (2023): 745-753.
